# Phytochemical Profiling, Biological Activities, and Development of Hydrogel Sheets Containing *Phanera sirindhorniae* Leaf Extract

**DOI:** 10.3390/gels12080729

**Published:** 2026-08-16

**Authors:** Chuda Chittasupho, Piyapong Pumival, Weerasak Samee, Sudarshan Singh, Julalak Chorachoo Ontong, Siriporn Okonogi, Nophadon Luangpirom, Marlyn Dian Laksitorini, Sirivan Athikomkulchai

**Affiliations:** 1 Faculty of Pharmacy, Chiang Mai University, Chiang Mai 50200, Thailand; chuda.c@cmu.ac.th (C.C.); sudarshan.s@cmu.ac.th (S.S.); okng2000@gmail.com (S.O.); 2 Center of Excellence in Pharmaceutical Nanotechnology, Faculty of Pharmacy, Chiang Mai University, Chiang Mai 50200, Thailand; 3 Sakaeo Crown Prince Hospital, Mueang Sa Kaeot, Sa Kaeo 27000, Thailand; piya101_p@hotmail.co.th; 4 Faculty of Pharmacy, Srinakharinwirot University, Nakhon Nayok 26120, Thailand; weerasak@g.swu.ac.th; 5 Office of Research Administration, Chiang Mai University, Chiang Mai 50200, Thailand; 6 Cosmetic Technology and Dietary Supplement Products Program, Faculty of Agro- and Bio-Industry, Thaksin University, Phatthalung Campus, Phatthalung 93210, Thailand; julalak.o@tsu.ac.th; 7 High-Value Bioproduct Innovation Research Center, Faculty of Agro- and Bio-Industry, Thaksin University, Phatthalung Campus, Phatthalung 93210, Thailand; 8 Faculty of Health Sciences, Shinawatra University, Pathum Thani 12160, Thailand; nophadon.l@siu.ac.th; 9 Faculty of Pharmacy, Universitas Gadjah Mada, Yogyakarta 55281, Indonesia; marlyn_fa@ugm.ac.id

**Keywords:** *Phanera sirindhorniae*, hydrogel sheet, topical drug delivery, plant based therapeutics, natural products

## Abstract

*Phanera sirindhorniae* (formerly known as *Bauhinia sirindhorniae*) is a Thai medicinal plant with reported traditional use for the treatment of inflammation; however, its comprehensive phytochemical, biological, and formulation potential remains underexplored. This study aimed to evaluate the phytochemical composition, antioxidant, anti-inflammatory, and antimicrobial activities of extracts from the flower (BSFE), stem (BSSE), and leaf (BSLE), and to develop a BSLE-loaded hydrogel sheet for topical application. Among the extracts, BSSE exhibited the highest total phenolic content (503.71 ± 7.56 mg GAE/g) and total flavonoid content (4778.67 ± 255.47 mg EGCG/g), along with strong antioxidant activity, as demonstrated by DPPH (IC_50_ = 17.05 µg/mL) and FRAP assays. BSLE showed superior anti-inflammatory activity by significantly suppressing nitric oxide and pro-inflammatory cytokines (TNF-α, IL-1β, and IL-6) in LPS-stimulated RAW264.7 macrophages, whereas BSSE displayed moderate effects. In antimicrobial evaluation, BSSE exhibited the strongest activity among the extracts, particularly against *Escherichia coli* and *Staphylococcus* species, although its potency remained lower than that of standard antibiotics. HPLC analysis identified 3,4-dihydroxybenzoic acid and luteolin as characteristic anti-inflammatory markers in BSLE. Based on its promising anti-inflammatory activity, BSLE was incorporated into hydrogel sheets (BSLE-F1 and BSLE-F2) using HPMC/PVA matrices with varying PEG 600 content. Both formulations demonstrated suitable physicochemical properties and skin-compatible pH. BSLE-F2 showed improved water retention and dimensional stability, whereas BSLE-F1 exhibited superior peelability, swelling capacity, and tensile strength. In conclusion, BSLE-F1 demonstrated a more favorable balance of mechanical and functional properties, suggesting its suitability as a topical anti-inflammatory hydrogel sheet. This study highlights the potential of *P. sirindhorniae* extracts for developing multifunctional, natural-based topical delivery systems.

## 1. Introduction

Plants used in traditional medicine remain an important source of bioactive compounds for pharmaceutical development, particularly in the search for new anti-inflammatory and antimicrobial agents suitable for topical delivery systems [1]. *Phanera sirindhorniae* (K.Larsen & S.S.Larsen) Mackinder & R.Clark, formerly classified as *Bauhinia sirindhorniae* K. & S.S. Larsen, is a traditional Thai medicinal plant locally known as *Sam Sip Song Pradong* [2]. It has traditionally been used to promote immune health, while an infusion of its stem has been employed for the treatment of inflammatory conditions. Early phytochemical investigations of its stems and roots led to the isolation of seventeen phenolic compounds, including flavonoids, chalcones, chromones, lignan glycosides, cyanogenic glycosides, and triterpenoids. Several of these metabolites, particularly (2S)-eriodictyol, isoliquiritigenin, luteolin, and (2S)-naringenin, exhibited antibacterial, antioxidant, and cytotoxic activities, supporting the plant’s ethnopharmacological relevance [3,4].

Previous studies demonstrated that ethanol extracts from the roots and stems inhibited *Bacillus subtilis* and *Staphylococcus aureus*, with minimum inhibitory concentrations (MICs) ranging from 50 to 200 µg/mL [5]. In addition, the root acetone extract inhibited *Aeromonas hydrophila*, with an MIC of 97.66 ppm [6]. However, despite these promising findings, information regarding the anti-inflammatory activity of different plant parts remains limited, and no studies have systematically compared the phytochemical composition and biological activities of the flower, stem, and leaf extracts [3].

Since inflammatory skin disorders are frequently associated with oxidative stress and microbial colonization, multifunctional natural products possessing antioxidant, anti-inflammatory, and antibacterial properties represent promising candidates for topical therapeutic development [7]. Furthermore, plant-derived polyphenols are particularly attractive for topical formulations because they can simultaneously reduce oxidative damage, suppress inflammatory responses, and inhibit microbial growth, thereby potentially promoting skin repair and improving therapeutic outcomes [8]. Hydrogel sheets have attracted increasing attention for topical drug delivery because they combine the high water content and biocompatibility of hydrogels with prolonged residence time and sustained release characteristics [9,10,11].

Different plant organs often contain distinct phytochemical profiles, which may result in substantial differences in their biological activities. Although previous studies have reported the phytochemical constituents and selected biological activities of *P. sirindhorniae*, comparative information on the phytochemical composition and biological activities of its flower, stem, and leaf remains limited. Furthermore, the potential of the plant extracts for incorporation into topical formulations has not been systematically explored. Accordingly, this study aimed to (i) compare the phytochemical composition and antioxidant activities of flower, stem, and leaf extracts of *P. sirindhorniae*; (ii) evaluate their anti-inflammatory and antibacterial activities using relevant in vitro models; (iii) identify characteristic phytochemical markers by HPLC analysis; and (iv) incorporate the extract exhibiting the most suitable anti-inflammatory profile into hydrogel sheets and characterize their preliminary physicochemical and mechanical properties. This integrated approach provides comparative information on the organ-dependent biological activities of *P. sirindhorniae* and establishes an initial formulation framework for further development of its leaf extract as a topical hydrogel prototype.

## 2. Results

### 2.1. Total Phenolic Content of P. sirindhorniae Extracts

**Figure 1:**
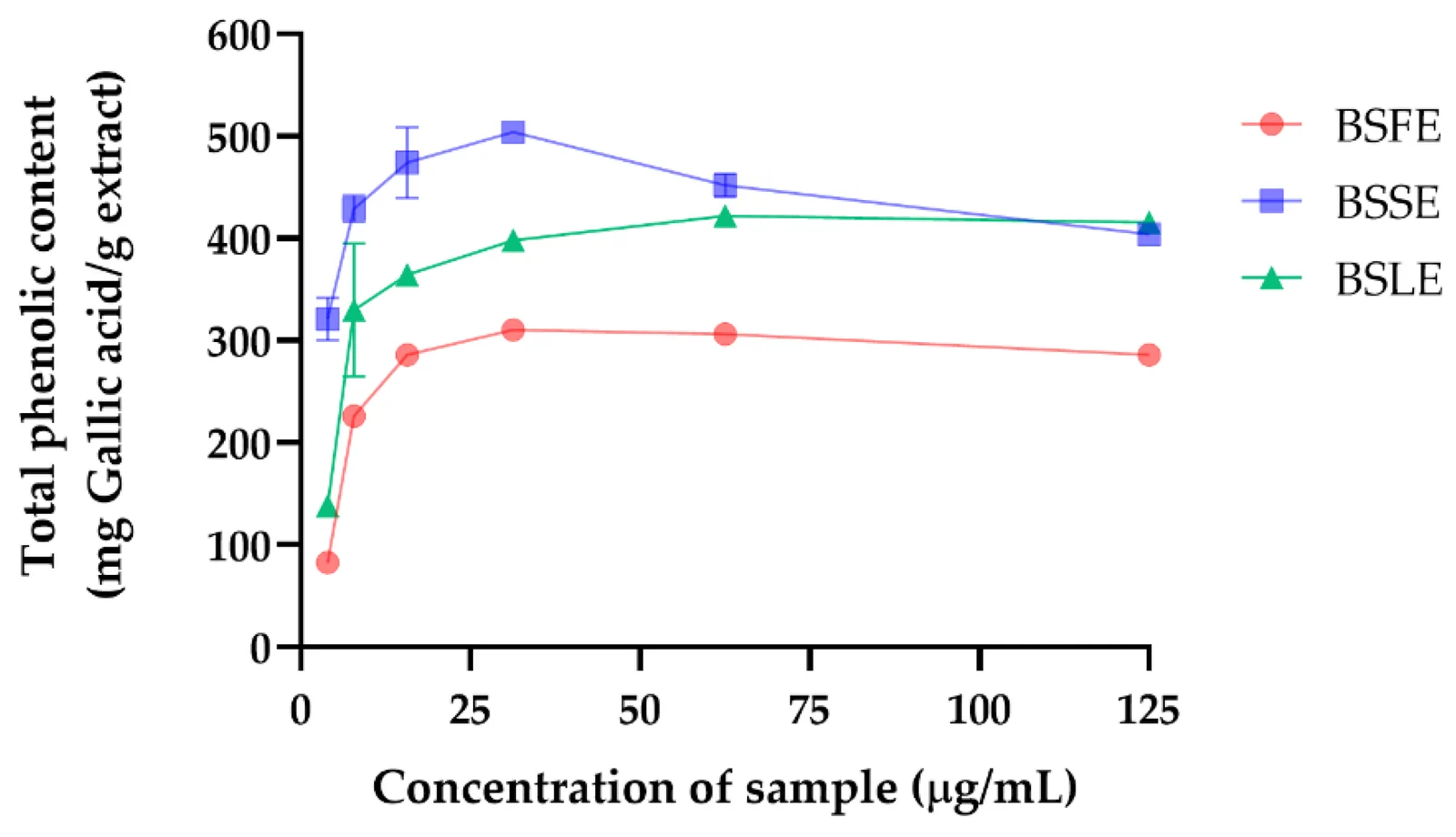
Total phenolic content (TPC) of BSFE, BSSE, and BSLE extracts. Data are presented as mean ± SD (n = 3).

A concentration-dependent increase in total phenolic content was observed for all extracts. BSSE demonstrated the greatest phenolic enrichment throughout the tested range, achieving a maximum value of 503.71 ± 7.56 mg GAE/g, after which a slight plateau was noted. BSLE exhibited a similar trend, with progressive elevation in TPC and a peak response of 422.11 ± 2.75 mg GAE/g. In contrast, BSFE showed a more gradual increase in phenolic content, reaching the lowest maximum among the samples at 310.40 ± 8.25 mg GAE/g. The graph confirms these trends, illustrating that while all extracts exhibited increasing phenolic content with concentration, BSSE consistently outperformed BSLE and BSFE, indicating a superior abundance of phenolic constituents (Figure 1).

### 2.2. Total Flavonoid Content of P. sirindhorniae Extracts

**Figure 2:**
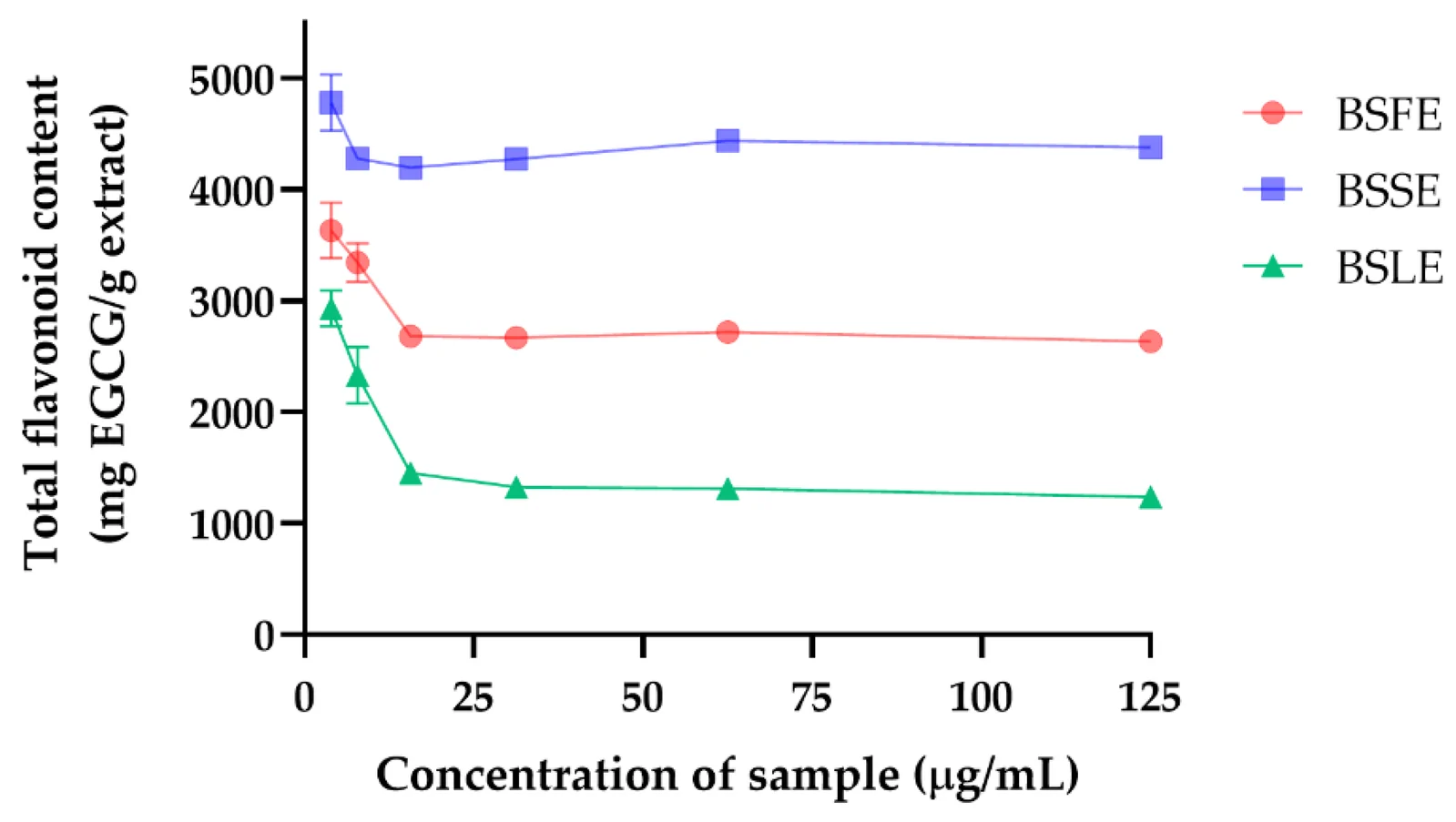
Total flavonoid content (TFC) of BSFE, BSSE, and BSLE extracts. Data are presented as mean ± SD (n = 3).

All extracts exhibited measurable flavonoid content, although the magnitude differed significantly among samples. BSSE contained the highest flavonoid levels, with a peak value of 4778.67 ± 255.47 mg EGCG/g extract, and remained relatively stable across concentrations. BSFE showed an intermediate TFC, decreasing gradually from 3631.41 ± 248.34 mg EGCG/g to 2632.89 ± 54.07 mg EGCG/g as the concentration increased. In contrast, BSLE demonstrated the lowest flavonoid abundance, declining sharply from 2929.78 ± 162.57 mg EGCG/g to 1234.37 ± 12.35 mg EGCG/g. The curve indicates that BSSE consistently exhibited the greatest flavonoid richness, followed by BSFE, whereas BSLE showed comparatively lower levels across the tested range (Figure 2).

### 2.3. DPPH Radical Scavenging Activity of P. sirindhorniae Extracts

**Figure 3:**
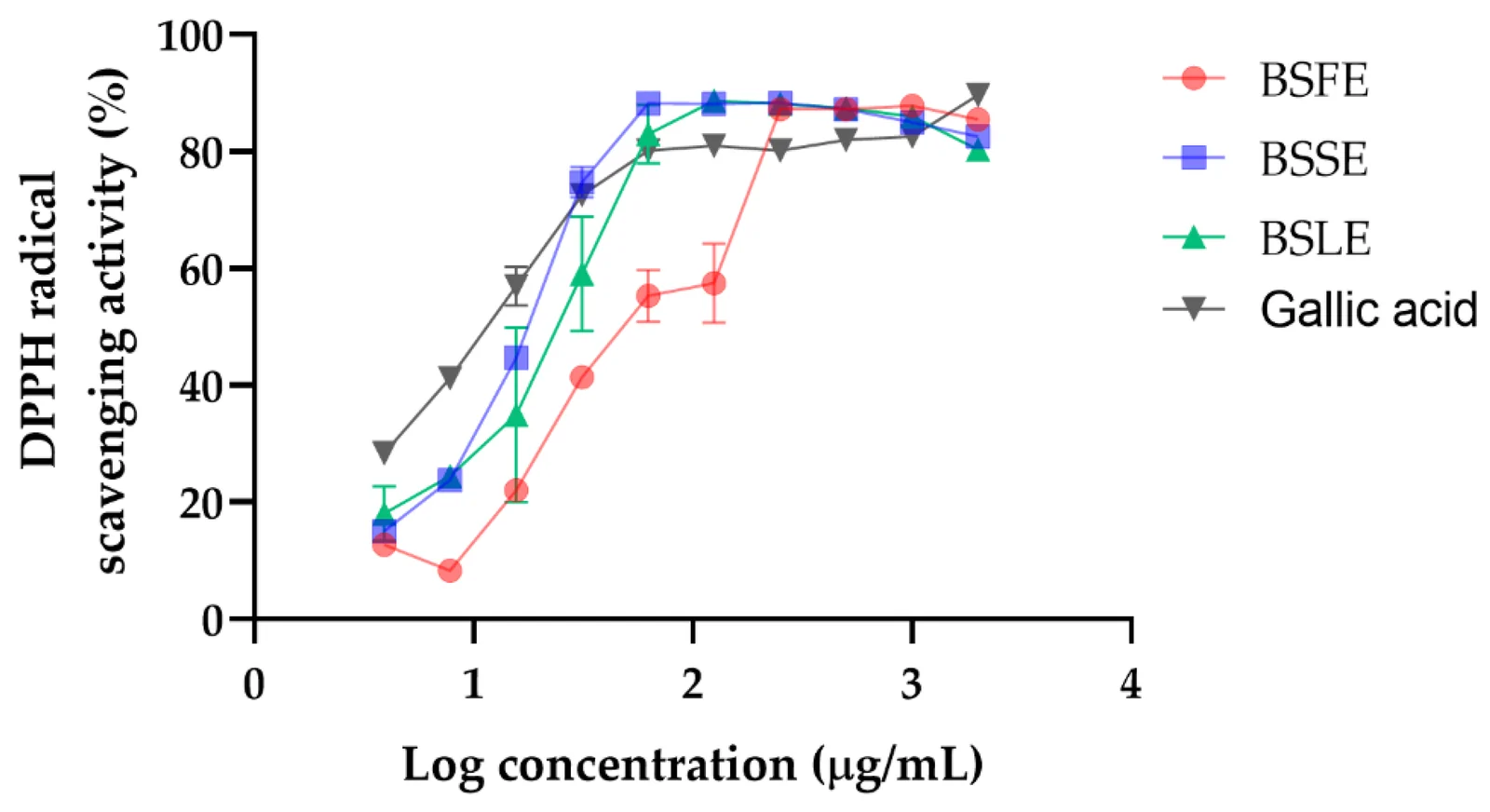
DPPH radical scavenging activity of BSFE, BSSE, and BSLE extracts compared with gallic acid. Data are presented as mean ± SD (n = 3).

All extracts exhibited a concentration-dependent enhancement in DPPH radical scavenging capacity. At lower concentrations, BSSE and BSLE demonstrated higher activity relative to BSFE, whereas gallic acid maintained superior scavenging efficiency across the same range. A marked increase in inhibition was observed as concentrations increased, with all samples achieving >80% scavenging at saturating levels. BSSE reached the highest maximal response (88.26 ± 0.17%), followed closely by BSLE (88.64 ± 0.43%) and BSFE (87.78 ± 0.23%), while gallic acid attained the greatest inhibition (89.63 ± 1.49%), consistent with its role as the reference antioxidant (Figure 3).

The potency ranking based on IC_50_ values further supported these findings. Gallic acid showed the strongest antioxidant activity (IC_50_ = 11.25 µg/mL), followed by BSSE (17.05 µg/mL), BSLE (22.30 µg/mL), and BSFE (58.10 µg/mL). These results indicate that BSSE had the highest radical-scavenging efficiency among the extracts, approaching that of the standard antioxidant gallic acid.

### 2.4. ABTS Radical Scavenging Activity of P. sirindhorniae Extracts

**Figure 4:**
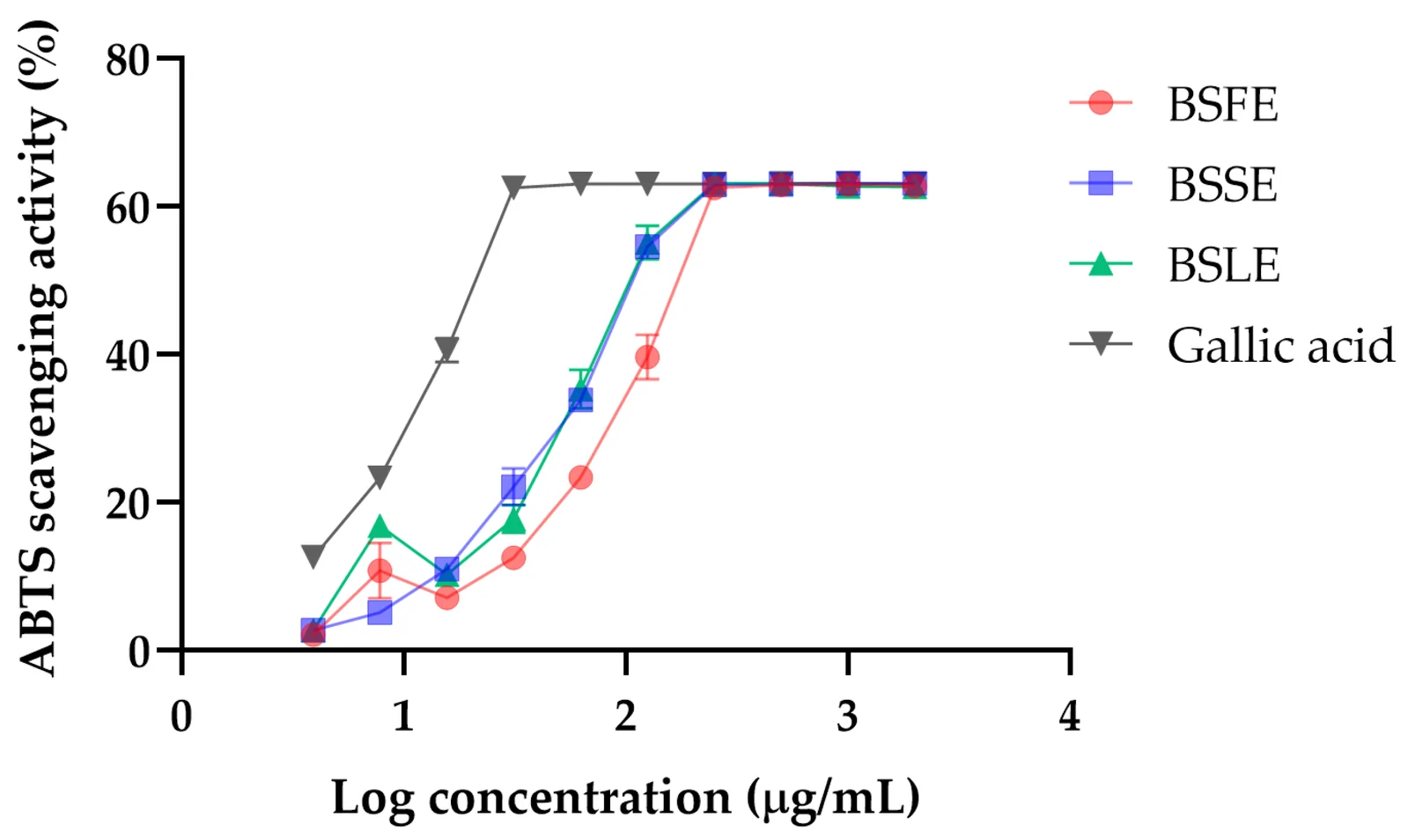
ABTS radical scavenging activity of BSFE, BSSE, and BSLE extracts compared with gallic acid. Data are shown as mean ± SD (n = 3).

All extracts exhibited dose-dependent increases in ABTS scavenging activity. At lower concentrations, gallic acid displayed the strongest scavenging capacity, followed by BSLE and BSSE, while BSFE showed the lowest activity. With increasing concentration, a steep rise in inhibition was observed across all samples, with maximal scavenging at higher concentrations (Figure 4). The potency ranking derived from IC_50_ values confirmed this trend: gallic acid exhibited the highest activity (IC_50_ = 62.18 µg/mL), followed by BSLE (IC_50_ = 226.8 µg/mL) and BSSE (IC_50_ = 228.4 µg/mL), while BSFE demonstrated the lowest ABTS scavenging efficiency (IC_50_ = 310.4 µg/mL). These findings indicate that although all extracts possessed measurable ABTS radical-scavenging activity, BSLE and BSSE were more effective than BSFE, but none matched the potency of gallic acid.

### 2.5. Ferric Reducing Antioxidant Power (FRAP) of P. sirindhorniae Extracts

**Figure 5:**
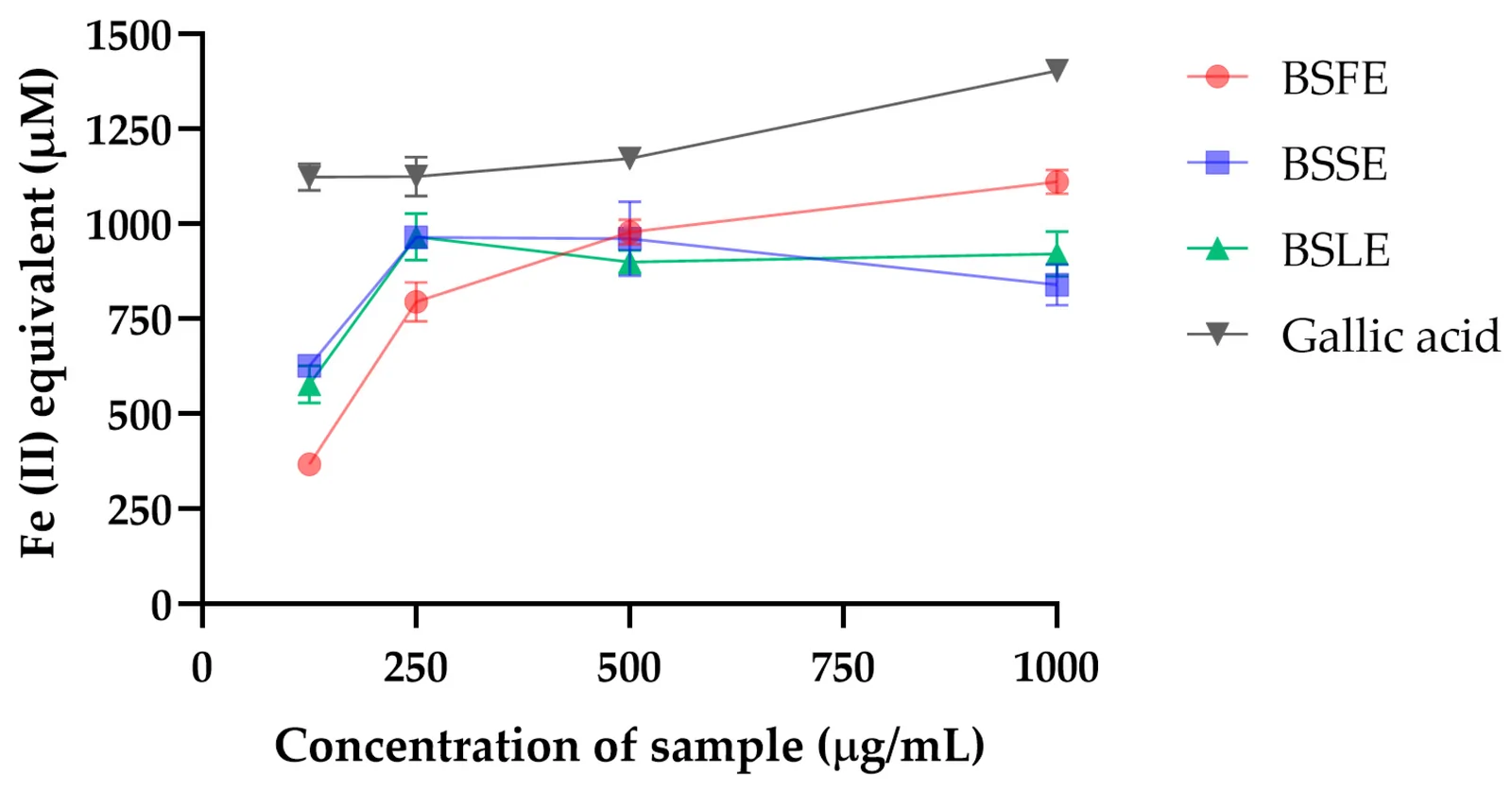
Ferric reducing antioxidant power (FRAP) of BSFE, BSSE, and BSLE extracts compared with gallic acid. Data are expressed as mean ± SD (n = 3).

All samples showed a concentration-dependent increase in ferric reducing capacity. At low concentrations, BSSE exhibited the strongest activity, followed closely by BSLE, while BSFE showed the lowest response. As concentrations increased, BSFE rose sharply and surpassed those of other extracts, yielding the highest FRAP value at 1000 µg/mL (1110.83 ± 30.89 µM Fe^2+^) (Figure 5). Throughout the range, gallic acid consistently demonstrated the greatest reducing power, culminating in 1403.50 ± 22.57 µM Fe^2+^ at the highest concentration.

### 2.6. Cytotoxicity of P. sirindhorniae Extracts

**Figure 6:**
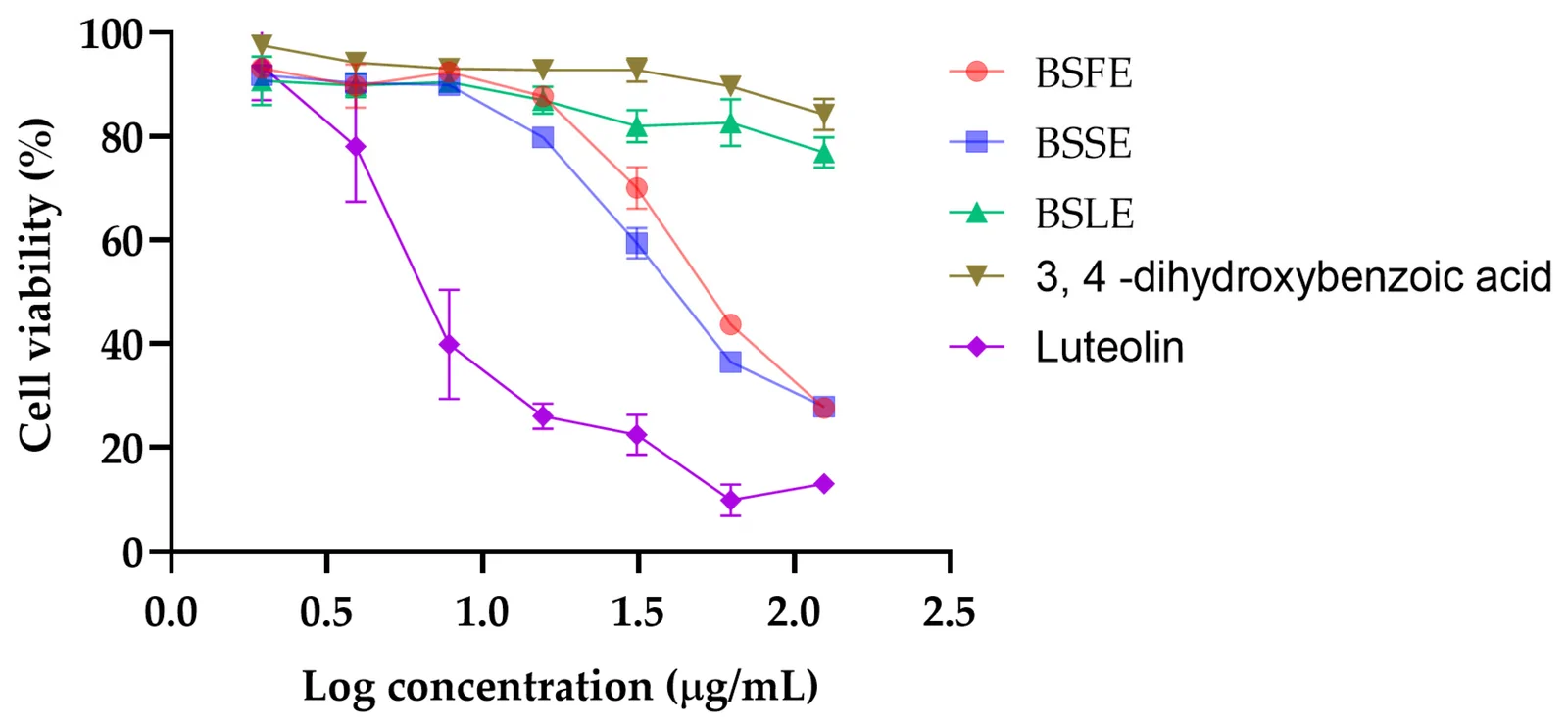
Effects of BSFE, BSSE, BSLE, 3,4-dihydroxybenzoic acid, and luteolin on RAW 264.7 cell viability. Data are presented as mean ± SD (n = 3).

All samples reduced RAW 264.7 cell viability in a concentration-dependent manner. At the lowest tested dose (1.95 µg/mL), BSFE, BSSE, and BSLE maintained high viability (93.10 ± 1.94%, 91.76 ± 1.13%, and 90.68 ± 4.68%, respectively), similar to 3,4-dihydroxybenzoic acid (97.57 ± 1.35%), while luteolin already reduced viability (93.57 ± 6.57%) (Figure 6). Calculated IC_50_ values supported these observations: luteolin displayed the highest cytotoxicity (IC_50_ = 8.05 µg/mL), followed by BSSE (44.99 µg/mL) and BSFE (56.73 µg/mL). In contrast, BSLE (IC_50_ = 10,347 µg/mL) and 3,4-dihydroxybenzoic acid (IC_50_ = 13,411 µg/mL) demonstrated negligible cytotoxicity toward RAW 264.7 cells.

### 2.7. Nitric Oxide Inhibition of P. sirindhorniae Extracts on LPS-Stimulated RAW 264.7 Macrophages

**Figure 7:**
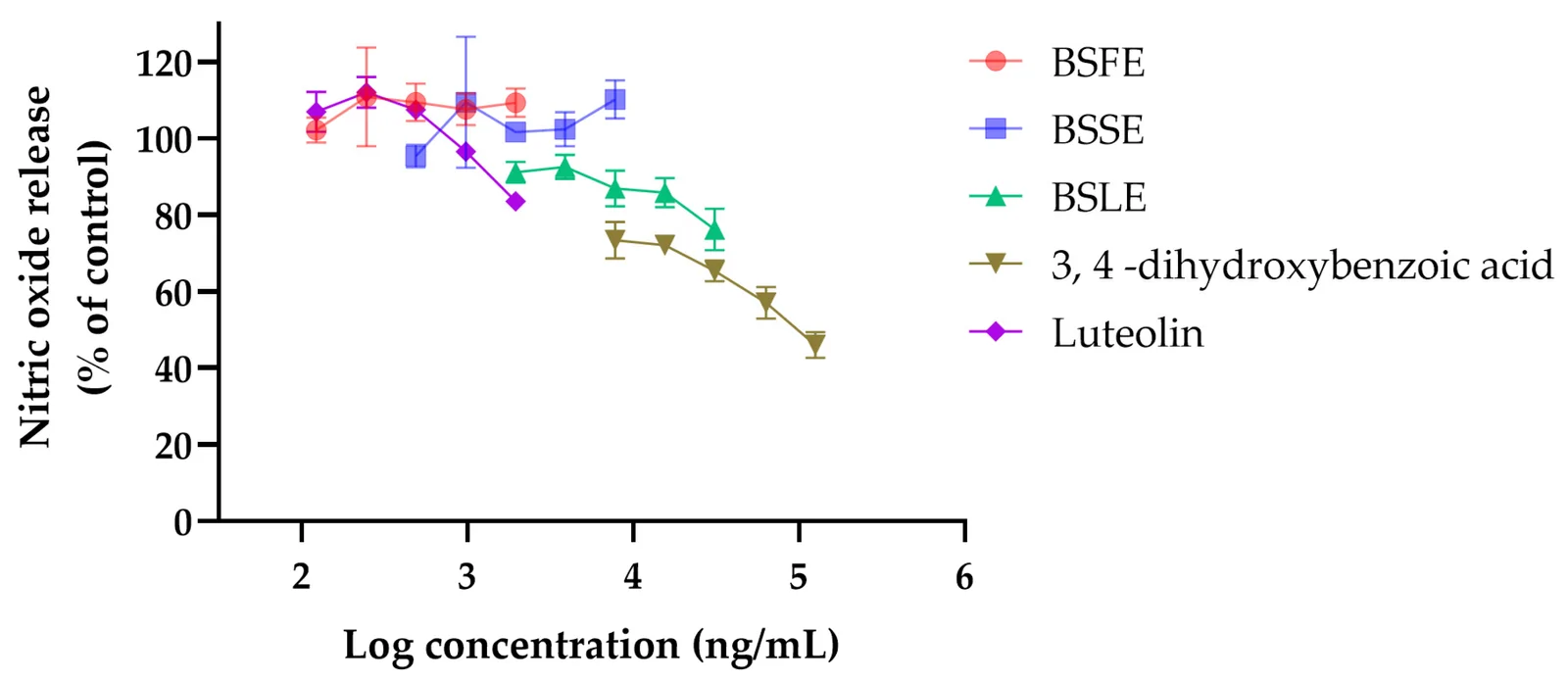
Nitric oxide (NO) inhibitory activity of BSFE, BSSE, BSLE, 3,4-dihydroxybenzoic acid, and luteolin in LPS-activated RAW 264.7 macrophages. Values are presented as mean ± SD (n = 3).

All extracts exhibited concentration-dependent suppression of LPS-induced nitric oxide (NO) production, although their inhibitory potency varied. BSLE showed progressive inhibition, reducing NO levels to 76.25 ± 5.49% at 1.95 µg/mL, whereas BSSE and BSFE produced only modest reductions across concentrations (Figure 7). Among reference compounds, 3,4-dihydroxybenzoic acid demonstrated strong inhibition, decreasing NO release to 45.99 ± 3.41% at 125 µg/mL, while luteolin exerted potent suppression, lowering NO to 83.55 ± 2.45% at the highest dose tested. The pharmacological control L-NAME confirmed this inhibitory trend, reducing NO in a dose-dependent manner from 93.33 ± 7.55% (20 µM) to 59.22 ± 0.78% (100 µM) and 39.67 ± 0.75% (200 µM), verifying assay responsiveness. These findings indicate that 3,4-dihydroxybenzoic acid exhibited the strongest NO-inhibitory activity, followed by luteolin and BSLE, whereas BSFE and BSSE showed weaker suppression, consistent with their lower anti-inflammatory potential relative to the standards and L-NAME.

### 2.8. Inhibition of TNF-α Production

**Figure 8:**
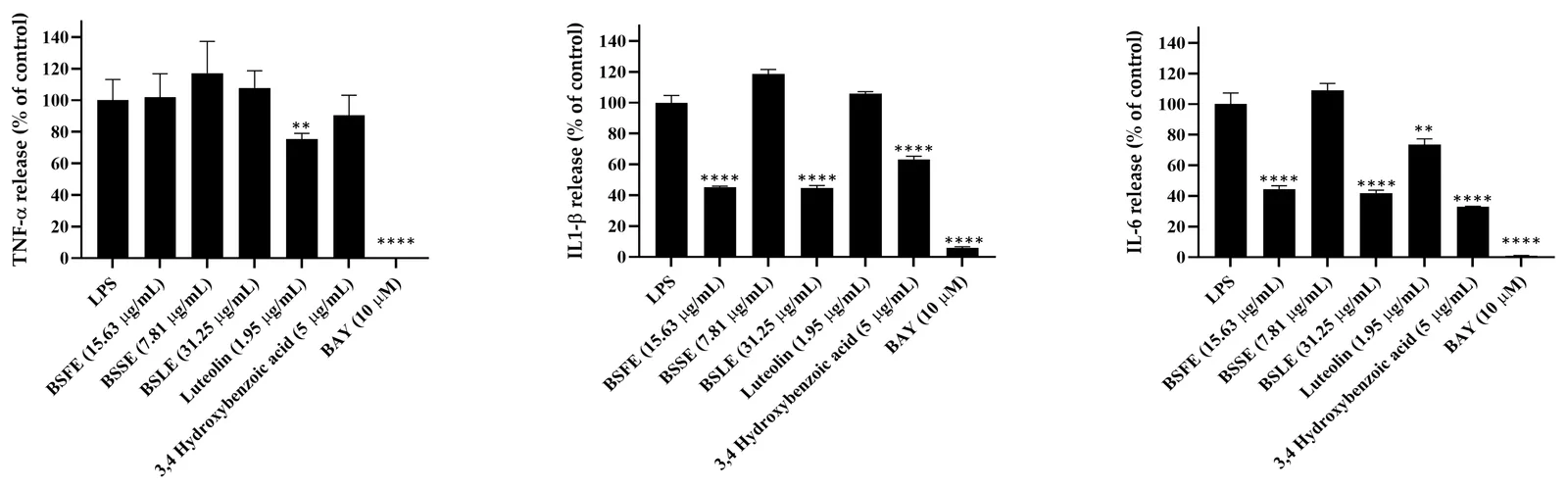
Suppression of pro-inflammatory cytokine production in LPS-activated RAW 264.7 macrophages following treatment with BSFE, BSSE, BSLE, luteolin, and 3,4-dihydroxybenzoic acid. Data are expressed as mean ± SD (n = 3). ** *p* < 0.01 and **** *p* < 0.0001 versus the LPS-treated control group.

TNF-α production in RAW 264.7 macrophages was normalized to the LPS-treated group (100%) (Figure 8). Treatment with BSFE (15.63 µg/mL), BSSE (7.81 µg/mL), and BSLE (31.25 µg/mL) did not suppress TNF-α production, with relative levels of 101.89 ± 14.95%, 117.14 ± 20.21%, and 107.84 ± 10.91%, respectively. Among the marker compounds, luteolin (1.95 µg/mL) significantly reduced TNF-α production to 75.38 ± 3.63%, whereas 3,4-hydroxybenzoic acid (5 µg/mL) produced only a modest, non-significant reduction (90.60 ± 12.59%). As expected, the positive control, BAY 11-7082 (10 µM), an NF-κB inhibitor, almost completely suppressed TNF-α production to 0.04 ± 0.01%, confirming the validity and responsiveness of the assay.

### 2.9. Inhibition of IL-1β Production

Treatment with BSFE (15.63 µg/mL) significantly reduced IL-1β production to 45.22 ± 0.72% of the LPS-treated group. Similarly, BSLE (31.25 µg/mL) markedly suppressed IL-1β production to 44.71 ± 1.54%, demonstrating the strongest inhibitory effect among the three extracts. In contrast, BSSE (7.81 µg/mL) did not inhibit IL-1β production, with the cytokine level increasing to 118.73 ± 2.78% relative to the LPS-treated group. Luteolin (1.95 µg/mL) showed no significant inhibitory effect, with IL-1β production remaining at 105.76 ± 1.44%, whereas 3,4-hydroxybenzoic acid (5 µg/mL) significantly reduced IL-1β production to 63.21 ± 2.08%. BAY 11-7082 (10 µM) markedly suppressed IL-1β production to 5.79 ± 0.88%.

### 2.10. Inhibition of IL-6 Production

BSFE (15.63 µg/mL) significantly reduced IL-6 production in RAW264.7 cells to 44.50 ± 2.25% of the LPS-treated group. BSLE (31.25 µg/mL) markedly suppressed IL-6 production to 41.93 ± 1.91%, demonstrating the strongest inhibitory effect among the three extracts. In contrast, BSSE (7.81 µg/mL) did not inhibit IL-6 production, with the cytokine level increasing to 108.94 ± 4.58% relative to the LPS-treated group. Luteolin (1.95 µg/mL) significantly reduced IL-6 production to 73.57 ± 3.71%, whereas 3,4-hydroxybenzoic acid (5 µg/mL) exhibited a stronger inhibitory effect, decreasing IL-6 production to 32.90 ± 0.43%. BAY 11-7082 (10 µM) completely suppressed IL-6 production to 0.99 ± 0.20%.

### 2.11. Antimicrobial Activity of P. sirindhorniae Extracts

All extracts exhibited antibacterial activity, although potency varied by organism and extract type. BSSE demonstrated the strongest antimicrobial effect, with MIC/MBC values of 128/128 µL/mL against *Escherichia coli* ATCC 25922, 128/>1024 µL/mL against *Pseudomonas aeruginosa* ATCC 27853, and 128/256 µL/mL against both *Staphylococcus aureus* ATCC 25923 and *Staphylococcus epidermidis* ATCC 35984. BSFE and BSLE showed weaker inhibition, with MIC values of 512 µL/mL for *E. coli*, *P. aeruginosa*, *S. aureus*, and *S. epidermidis*, and corresponding MBCs ranging from 512 to >1024 µL/mL.

Standard antibiotics exhibited markedly greater potency than plant extracts. Gentamicin exhibited MIC/MBC values of 0.5/0.5 µL/mL against *E. coli*, 0.5/1 µL/mL against *P. aeruginosa*, and Vancomycin showed MIC/MBC values of 0.5/0.5 µL/mL and 0.5/1 µL/mL against *S. aureus* and *S. epidermidis*, respectively. These results confirm that although BSSE exhibited the best antimicrobial activity among the extracts, its effectiveness remained substantially lower than that of conventional antibiotics.

### 2.12. Quantitative HPLC Profiling of 3,4-Dihydroxybenzoic Acid and Luteolin in BSLE

**Figure 9:**
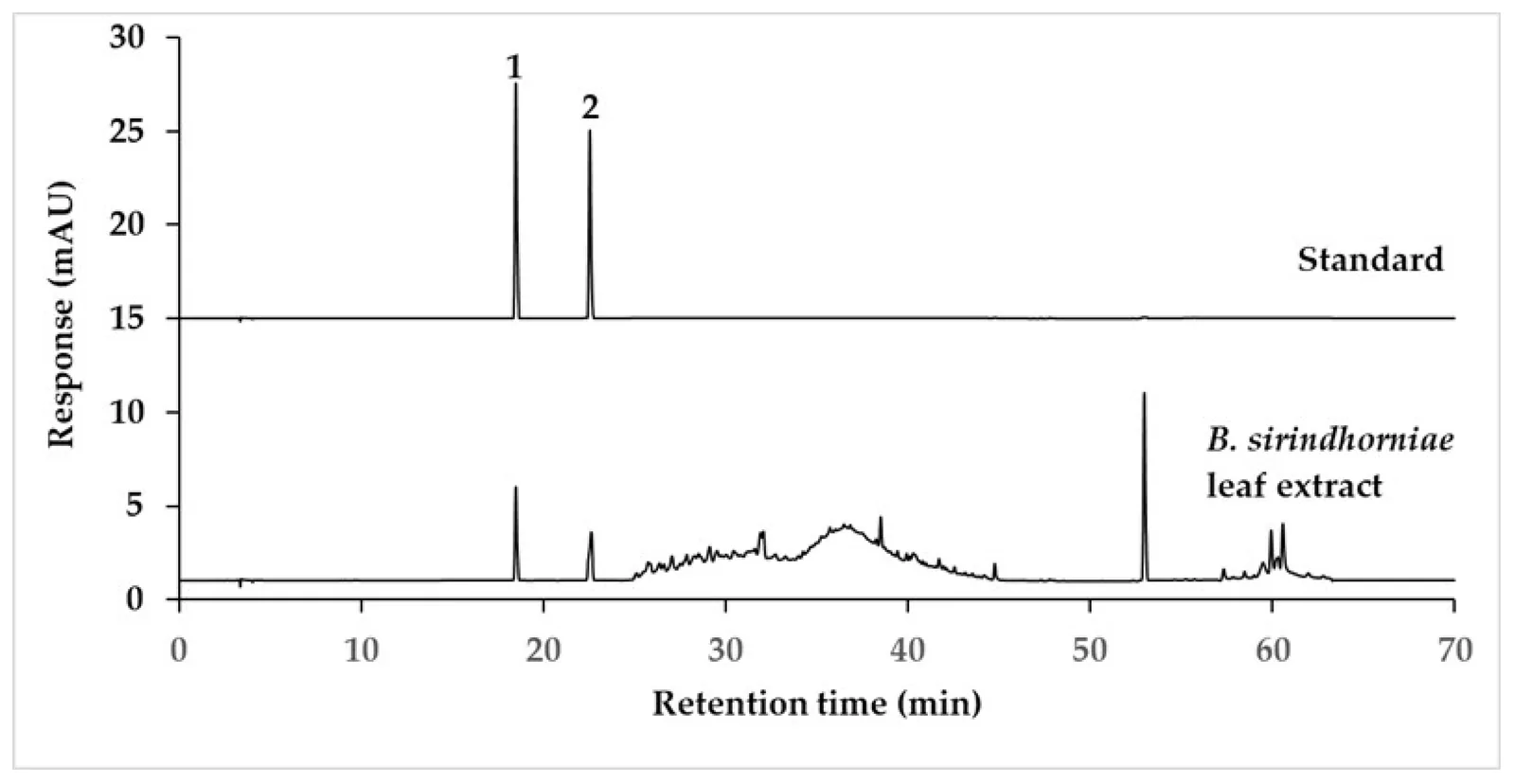
Chromatograms of 3,4-dihydroxybenzoic acid (1), luteolin (2), and BSLE, observed at a UV wavelength of 295 nm.

HPLC analysis successfully separated the two target phenolic compounds, 3,4-dihydroxybenzoic acid and luteolin, with retention times of 18.57 min and 22.59 min, respectively (Figure 9). In the standard chromatogram, peaks corresponding to 3,4-dihydroxybenzoic acid (peak 1) and luteolin (peak 2) were clearly observed, whereas the BSLE chromatogram showed multiple phytochemical peaks, including detectable peaks at the same retention times as the reference standards.

Quantification was performed using peak areas obtained from triplicate injections. For 3,4-dihydroxybenzoic acid, sample peak areas ranged from 48.14 to 50.67 µg/mL. The calculated content in the crude extract was 9.63, 9.87, and 10.13 µg/mg, yielding an average of 9.88 ± 0.25 µg/mg.

For luteolin, sample peak areas ranged from 4.46 to 4.72 µg/mL. The crude extract contained 0.89, 0.93, and 0.94 µg/mg, yielding a mean luteolin content of 0.92 ± 0.03 µg/mg extract. The results suggested that 3,4-dihydroxybenzoic acid was the major quantified phenolic compound in BSLE, present at approximately tenfold higher levels than luteolin.

### 2.13. BSLE-Loaded Hydrogel Sheets

#### 2.13.1. Visual Appearance and Macroscopic Stability

**Figure 10:**
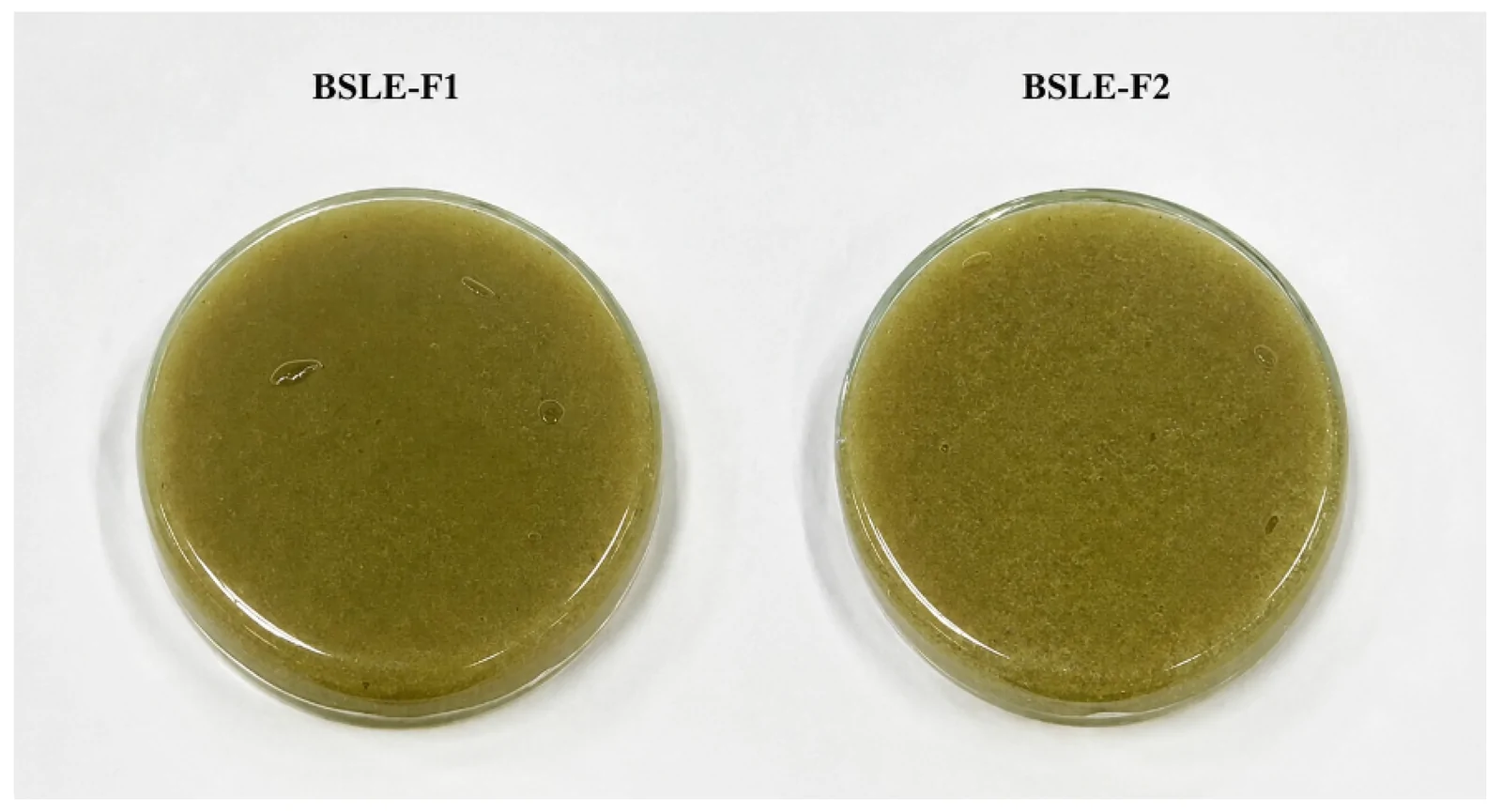
BSLE-loaded hydrogel sheet formulations, BSLE-F1 and BSLE-F2, immediately after preparation.

Both BSLE-F1 (2 g PEG 600) and BSLE-F2 (6 g PEG 600) were successfully prepared by the solvent-casting method (Figure 10). On Day 0, both formulations formed uniform, greenish-opaque hydrogel sheets with smooth surfaces, a soft, non-tacky texture, and no visible bubbles or cracks. After 7 days of storage at 4 °C, hydrogel sheets from both formulations exhibited a loss of structural integrity, with visible exudation of water and partial liquefaction, consistent with low-temperature syneresis. In contrast, hydrogel sheets stored at 30 and 45 °C retained their sheet-like appearance without phase separation, indicating better physical stability at ambient and elevated temperatures.

#### 2.13.2. pH

The initial pH of both BSLE-F1 and BSLE-F2 dispersions was 5.0 before and after oven drying. After 7 days of storage at 4, 30, and 45 °C, the pH of all samples remained at 5.0, indicating that short-term exposure to these temperatures did not alter the hydrogel sheet’s acidity and that the formulations are within a skin-compatible pH range.

#### 2.13.3. Water-Loss Index

**Figure 11:**
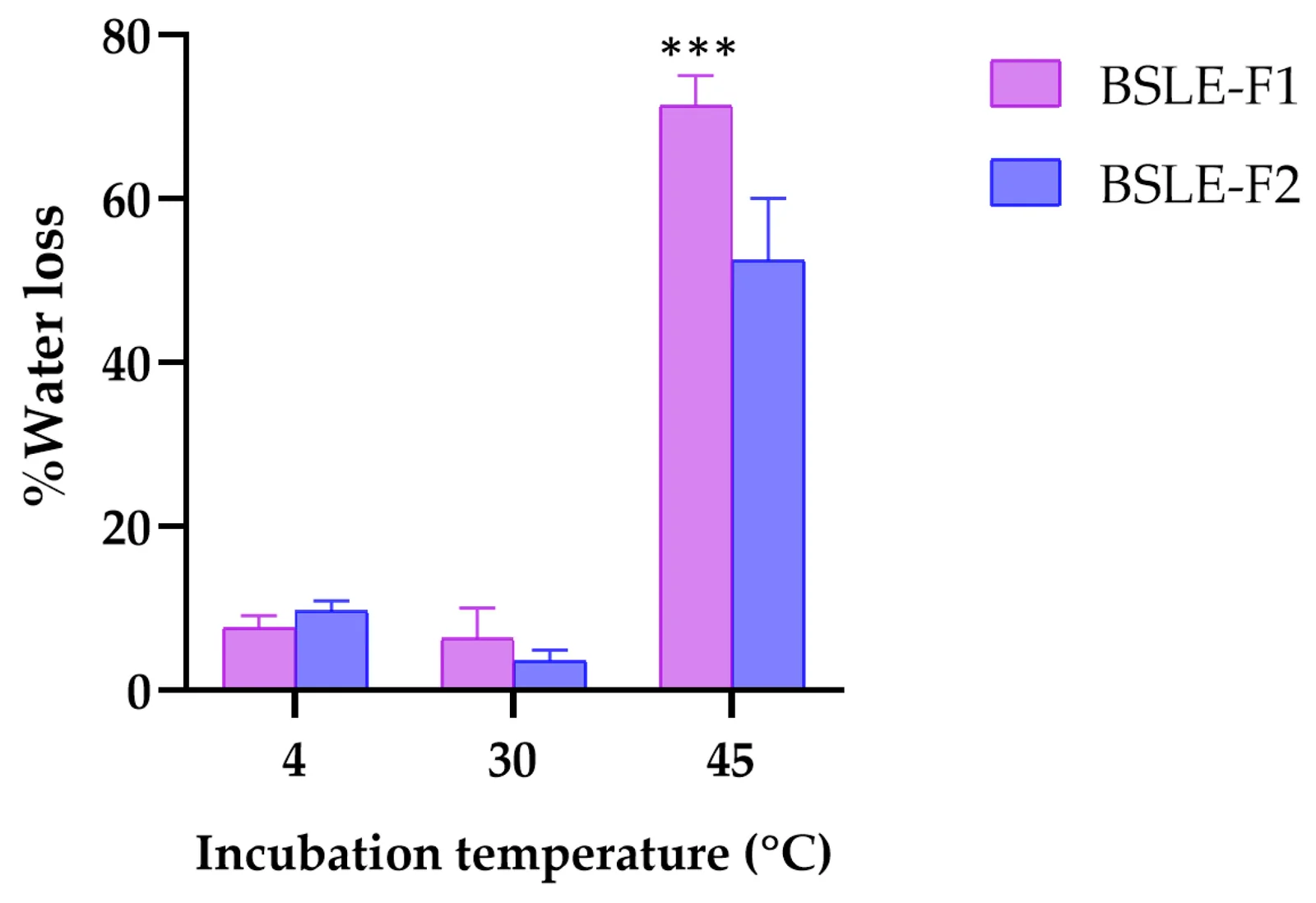
Water-loss percentages of BSLE-loaded hydrogel sheets (BSLE-F1 and BSLE-F2) after 7 days of storage at different temperatures (4, 30, and 45 °C). Data are presented as mean ± SD (n = 3). BSLE-F1 exhibited significantly greater water loss than BSLE-F2 at 45 °C (*** *p* < 0.001).

For BSLE-F1, the mean percentage water loss after 7 days was 7.64 ± 1.49%, 6.30 ± 3.73%, and 71.35 ± 3.66% at 4, 25, and 45 °C, respectively (Figure 11). For BSLE-F2, the corresponding values were 9.63 ± 1.24%, 3.56 ± 1.37%, and 52.40 ± 7.65%. At 4 °C, water loss did not differ significantly between the two hydrogel sheets. At 30 and 45 °C, BSLE-F2 showed numerically lower water loss, with the difference statistically significant at 45 °C (*p* < 0.05).

#### 2.13.4. Hydrogel Sheet Thickness

**Figure 12:**
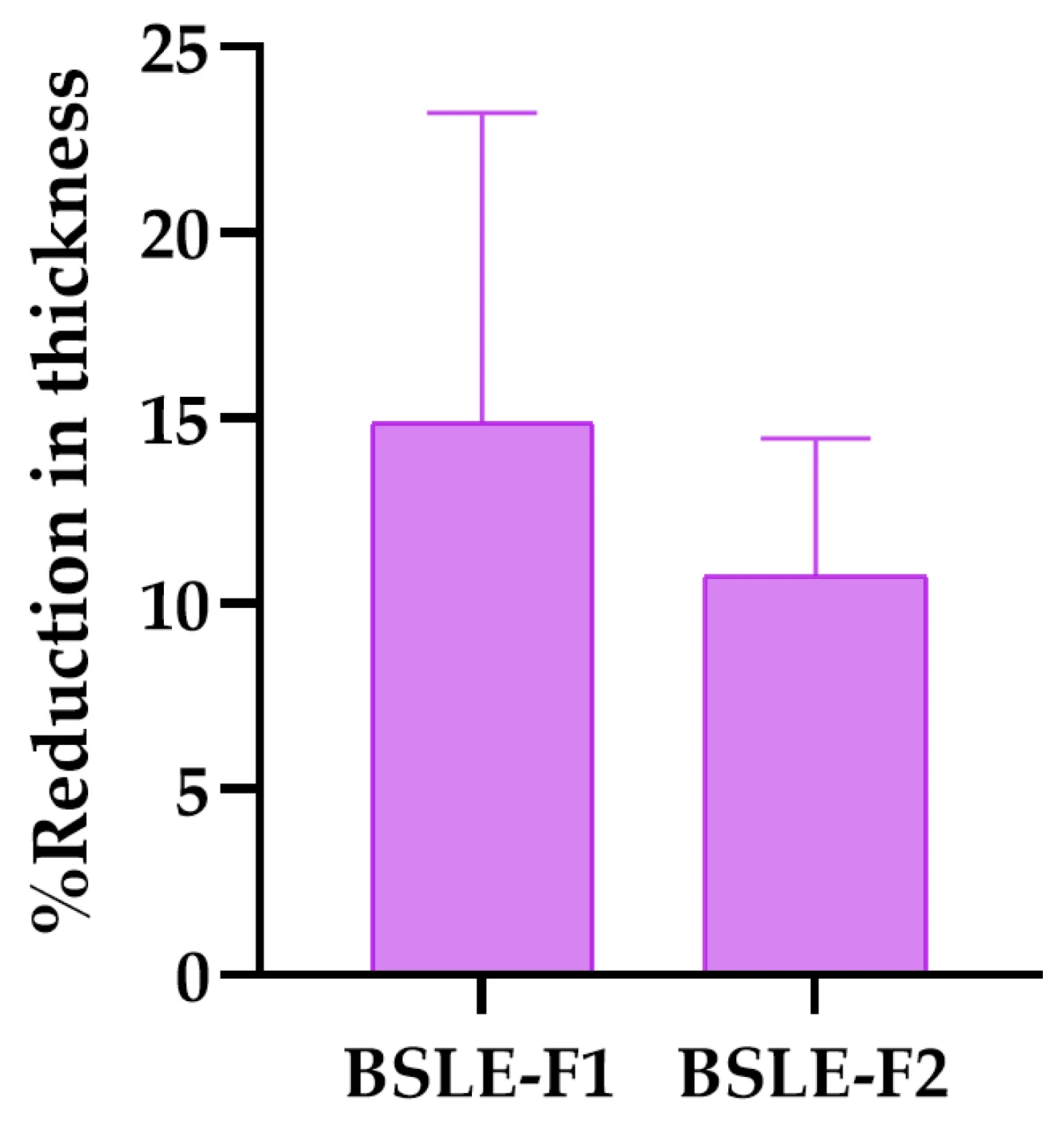
Percentage reduction in hydrogel sheet thickness of BSLE-loaded hydrogel sheets (BSLE-F1 and BSLE-F2) after 7 days of storage. Data are presented as mean ± SD (n = 3).

Both BSLE-F1 and BSLE-F2 hydrogel sheets exhibited a measurable reduction in thickness after 7 days of storage. At Day 0, the initial sheet thickness was approximately 3.0 mm for BSLE-F1 and 2.8–3.0 mm for BSLE-F2. Following storage, BSLE-F1 showed a greater percentage reduction in thickness (31.12%) compared with BSLE-F2 (10.74%), as illustrated in Figure 12. Although BSLE-F2 appeared more dimensionally stable, statistical analysis showed no significant difference in thickness reduction between the two formulations.

#### 2.13.5. Washability

The washability of BSLE-F1 and BSLE-F2 was evaluated by measuring the time required to remove residual formulation from the application surface after 1 h of contact. At Day 0, BSLE-F1 was removed immediately (0 s), whereas BSLE-F2 required 3.27 ± 2.89 s for complete removal. After 7 days of storage at 4 °C, the washing time increased to 17.11 ± 3.31 s for BSLE-F1 and 14.03 ± 4.94 s for BSLE-F2. In contrast, after storage at 30 °C, BSLE-F1 required 7.96 ± 2.83 s for removal, while BSLE-F2 was removed immediately. Following storage at 45 °C, both formulations were removed immediately (0 s). The results suggested that storage temperature influenced the washability of the hydrogel sheets, with the longest washing times observed after storage at 4 °C.

#### 2.13.6. Peel Ability and Residual Gel

The peelability of the BSLE-loaded hydrogel sheets was evaluated by determining the percentage of gel residue remaining on the plastic substrate after sheet removal. The results demonstrated that BSLE-F1 exhibited a significantly lower residue (0.046 ± 0.048%) compared to BSLE-F2 (0.143 ± 0.099%).

The lower residual gel observed in BSLE-F1 indicates superior peel ability and easier detachment from the substrate. This characteristic is particularly important for topical applications, as it suggests improved user comfort and minimal residue remaining on the skin after removal. In contrast, the higher residue observed in BSLE-F2 may be attributed to its higher PEG 600 content, which can increase the formulation’s viscosity and adhesive properties.

#### 2.13.7. Swelling Index

The swelling behavior of the prepared hydrogel sheets was evaluated by immersing the sheets in distilled water and monitoring the percentage increase in weight over time. Both formulations exhibited time-dependent swelling; however, BSLE-F1 consistently showed a higher swelling index than BSLE-F2 at all observed time points.

At 5 min, the swelling indices of BSLE-F1 and BSLE-F2 were 7.25 ± 2.71% and 4.25 ± 2.09%, respectively. The swelling capacity increased substantially at 15 min, reaching 19.59 ± 3.73% for BSLE-F1 and 10.44 ± 5.43% for BSLE-F2. At 30 min, BSLE-F1 achieved a swelling index of 22.56 ± 3.14%, whereas BSLE-F2 reached 15.74 ± 7.87%.

#### 2.13.8. Mechanical Properties

The mechanical properties of the hydrogel sheets were assessed using tensile strength. BSLE-F1 exhibited a tensile strength of 0.00173 ± 0.000071 N/mm^2^, which was slightly higher than that of BSLE-F2 (0.00150 ± 0.000097 N/mm^2^).

## 3. Discussion

The present study demonstrates that different parts of *P. sirindhorniae* possess distinct biological activities despite originating from the same plant. Among the three extracts, BSSE exhibited the highest total phenolic and flavonoid contents together with the strongest antioxidant and antibacterial activities, whereas BSLE consistently showed the greatest anti-inflammatory activity with minimal cytotoxicity. These findings indicate that the pharmacological properties of *P. sirindhorniae* are highly dependent on the plant organ used for extraction and suggest that different tissues accumulate distinct profiles of bioactive secondary metabolites [12].

The superior antioxidant activity of BSSE corresponded well with its high total phenolic and flavonoid contents. Phenolic compounds are recognized as efficient hydrogen- and electron-donating molecules capable of neutralizing reactive oxygen species and interrupting free-radical chain reactions [13]. Consequently, the higher phenolic abundance in the stem extract likely contributed to its lower DPPH IC_50_ value and greater reducing capacity compared with the flower and leaf extracts. Similar positive correlations between total phenolic content and antioxidant capacity have been widely reported in medicinal plants, although antioxidant activity is also influenced by the structural characteristics and relative abundance of individual phenolic constituents rather than total phenolic concentration alone [14,15,16].

Interestingly, antioxidant capacity did not directly correlate with anti-inflammatory activity in the present study. Although BSSE exhibited the highest total phenolic and flavonoid contents together with superior antioxidant activity, it showed only weak inhibition of nitric oxide production and did not suppress the pro-inflammatory cytokines IL-1β and IL-6; rather, their levels were increased relative to the LPS-treated control under the experimental conditions. This unexpected response indicates that high total phenolic and flavonoid contents alone cannot be interpreted as evidence of anti-inflammatory activity [17]. Total phenolic and flavonoid assays provide an estimate of the overall abundance of these compound classes but do not distinguish individual constituents with potentially different, or even opposing, biological effects. Therefore, the contrasting responses of BSSE and BSLE may reflect differences in their specific phytochemical profiles, relative concentrations of individual constituents, and interactions among multiple compounds rather than differences in total phenolic or flavonoid content alone. However, the present study did not characterize the constituents responsible for the cytokine response observed with BSSE or investigate the underlying signaling mechanisms; therefore, a mechanistic explanation cannot be established from the current data. Further phytochemical characterization and pathway-specific studies are warranted to determine the constituents and mechanisms responsible for this response.

Plant-derived secondary metabolites can differentially modulate macrophage inflammatory responses through multiple signaling pathways, including NF-κB, MAPKs, AP-1, STATs, and PI3K/AKT [18]. 3,4-Dihydroxybenzoic acid has been reported to suppress LPS-induced inflammatory responses by inhibiting the production of NO, TNF-α, IL-1β, and PGE_2_ through downregulation of iNOS and COX-2 expression. These effects are primarily mediated via inhibition of the NF-κB and MAPK signaling pathways [19]. Likewise, luteolin is a well-established anti-inflammatory flavonoid that inhibits the production of NO, TNF-α, IL-1β, IL-6, and PGE_2_ by suppressing multiple inflammatory signaling pathways, including NF-κB, MAPKs (ERK, JNK, and p38), and STAT3, thereby reducing the expression of iNOS and COX-2 [20]. Consistent with this mechanism, the purified 3,4-dihydroxybenzoic acid standard exhibited the strongest inhibition of nitric oxide, IL-1β, and IL-6 production among the tested phytochemicals, supporting its contribution to the anti-inflammatory activity of BSLE. Nevertheless, the detection of 3,4-dihydroxybenzoic acid and luteolin in BSLE does not establish that these compounds are solely responsible for the activity of the crude extract, and possible contributions or interactions of other constituents cannot be excluded. Thus, the present findings support an organ-dependent difference in the biological activity of *P. sirindhorniae*, while further mechanistic studies are required to establish the phytochemical basis of these differences.

The antibacterial activity observed among the extracts further supports the organ-specific distribution of bioactive compounds within *P. sirindhorniae*. BSSE demonstrated the lowest MIC and MBC values against both Gram-positive and Gram-negative bacteria, suggesting that antimicrobial constituents are enriched in the stem tissue. Phenolic compounds and flavonoids have been reported to inhibit bacterial growth through multiple mechanisms, including disruption of membrane integrity, inhibition of nucleic acid synthesis, interference with bacterial enzymes, and induction of oxidative damage [21]. This mechanism is consistent with the strong antibacterial activity observed for BSSE, which contained the highest total phenolic and flavonoid contents among the three extracts. The higher antibacterial potency of BSSE is therefore consistent with its greater abundance of phenolic compounds. Nevertheless, the antibacterial activity of all extracts remained substantially lower than that of conventional antibiotics, indicating that these botanical extracts are unlikely to function as stand-alone antimicrobial agents. Therefore, the observed antibacterial activity should be regarded as a complementary biological property, while the anti-inflammatory activity of BSLE represents the primary rationale for its selection for topical formulation development.

Although BSSE exhibited stronger antioxidant and antibacterial activities, BSLE was selected for hydrogel formulation because anti-inflammatory activity was the primary biological consideration for the intended topical application. BSLE showed the most favorable anti-inflammatory profile together with low cytotoxicity, whereas BSSE showed only weak inhibition of nitric oxide production and did not suppress IL-1β or IL-6 under the experimental conditions. Hydrogel sheets provide several pharmaceutical advantages over conventional semisolid formulations, including prolonged residence time at the application site, formation of a protective barrier, sustained release of bioactive compounds, and improved patient compliance [22]. Following solvent evaporation, these systems form thin polymeric sheets that conform closely to the skin surface while maintaining a moist microenvironment favorable for tissue repair and inflammation control [23].

The principal difference between BSLE-F1 and BSLE-F2 was the concentration of PEG 600, which served as a plasticizer. Plasticizers reduce intermolecular interactions among polymer chains, increasing chain mobility and flexibility while simultaneously influencing water retention, swelling behavior, mechanical strength, and hydrogel sheet adhesion [24]. BSLE-F2 exhibited significantly lower water loss than BSLE-F1 under the 45 °C short-term stress condition, indicating superior water-retention capacity under thermal stress. The 45 °C condition was included as a preliminary accelerated stress test to compare the relative physical responses of the two formulations and was not intended to represent routine storage conditions or a formal pharmaceutical stability study. Nevertheless, the substantial water loss observed for both formulations at 45 °C, particularly BSLE-F1, indicates limited physical resistance to elevated-temperature stress and highlights the need for further formulation optimization. The hygroscopic nature of PEG 600 enables extensive hydrogen bonding with water molecules, thereby reducing moisture evaporation and maintaining hydration within the polymer matrix [25]. Similar effects have been widely reported in HPMC- and PVA-based sheet-forming systems, where increasing PEG concentration improves moisture retention but simultaneously softens the polymer network [26].

Conversely, the lower PEG concentration in BSLE-F1 resulted in greater swelling capacity, improved peelability, and slightly higher tensile strength. Reduced plasticizer content allows stronger intermolecular interactions between polymer chains, producing a more cohesive network that better resists mechanical deformation while maintaining adequate flexibility [27]. The higher swelling index observed in BSLE-F1 may reflect a less densely packed polymer structure, allowing greater penetration of aqueous media into the hydrogel network. Such swelling behavior may be advantageous for maintaining hydration at the application site; however, its influence on phytochemical release and skin permeation requires further investigation [28].

Both formulations were readily removable with water, although washing time varied with storage temperature. The longest washing times were observed after storage at 4 °C, whereas shorter washing times were generally observed after storage at higher temperatures. This variation may be associated with temperature-dependent changes in the HPMC matrix, as aqueous HPMC systems exhibit thermoreversible sol–gel behavior, and their hydration, swelling, and dissolution characteristics are influenced by temperature and the composition of the surrounding medium [29].

The peelability results further demonstrated the influence of PEG concentration on hydrogel sheet performance. BSLE-F1 left significantly less residual gel following removal, indicating cleaner detachment from the application surface. In contrast, the higher PEG content of BSLE-F2 increased sheet adhesiveness, resulting in greater residue after peeling. Although stronger adhesion may prolong contact with the skin, excessive residue may reduce patient convenience and acceptance, particularly for cosmetic or repeated therapeutic applications. Therefore, an appropriate balance between adhesion and removability is essential during formulation development. Interestingly, despite exhibiting greater water loss under the 45 °C short-term stress condition, BSLE-F1 maintained comparable dimensional stability and slightly higher tensile strength than BSLE-F2 [30].

Collectively, these findings demonstrate that PEG 600 significantly influenced the physicochemical properties of the hydrogel sheets. Increasing PEG concentration improved water retention but reduced swelling capacity and mechanical strength while increasing hydrogel sheet adhesiveness. Although no significant difference in thickness reduction was observed between BSLE-F1 and BSLE-F2, BSLE-F1 exhibited greater peelability and swelling capacity together with slightly higher mechanical strength and a skin-compatible pH. Thus, the assessment of BSLE-F1 was based on its overall physicochemical and mechanical performance rather than dimensional stability alone. However, both formulations exhibited physical instability at 4 °C, while substantial water loss was observed under the 45 °C short-term stress condition. Therefore, the present 7-day evaluation should be considered a preliminary assessment of short-term physicochemical stability rather than a formal shelf-life study. Further formulation optimization and longer-term stability studies under appropriate storage conditions are required to establish product stability and shelf life before practical application. In addition, in vitro release and skin permeation studies are needed to determine the release of the incorporated phytochemicals and their ability to reach the intended site of topical action.

## 4. Conclusions

This study demonstrated that different parts of *P. sirindhorniae* possess distinct biological activities. BSSE exhibited the strongest antioxidant and antibacterial activities, whereas BSLE showed superior anti-inflammatory activity, with 3,4-dihydroxybenzoic acid and luteolin identified as potential contributing phytochemicals. Based on its anti-inflammatory activity and low cytotoxicity, BSLE was selected for incorporation into hydrogel sheets as a preliminary topical formulation prototype. Among the formulations, BSLE-F1 showed greater peelability, swelling capacity, and mechanical strength, while remaining physically acceptable under ambient storage conditions during the short-term evaluation; however, refrigeration induced syneresis, and substantial water loss occurred under elevated-temperature stress conditions. These findings provide an initial basis for further development of BSLE-loaded hydrogel sheets. Further formulation optimization, long-term stability evaluation, in vitro release, skin permeation, and in vivo efficacy studies are required to establish their suitability for topical application.

## 5. Materials and Methods

### 5.1. Materials

Gallic acid, epigallocatechin gallate (EGCG; ≥98%), Griess reagent, 2,2-diphenyl-1-picrylhydrazyl (DPPH), 2,4,6-tris(2-pyridyl)-s-triazine (TPTZ), and 2,2′-azino-bis(3-ethylbenzothiazoline-6-sulfonic acid) (ABTS) were sourced from Sigma-Aldrich (St. Louis, MO, USA). Absolute ethanol, dimethyl sulfoxide (DMSO), sodium bicarbonate, sodium nitrate, acetic acid, and sodium hydroxide were obtained from RCI Labscan (Bangkok, Thailand). Iron(III) chloride hexahydrate and hydrochloric acid (37%) were supplied by Qrec (Auckland, New Zealand), whereas Folin–Ciocalteu reagent, aluminum chloride, sodium acetate trihydrate, ferrous sulfate heptahydrate (99%), and potassium persulfate were obtained from Loba Chemie (Mumbai, India). Cell culture reagents, including high-glucose Dulbecco’s Modified Eagle’s Medium (DMEM), fetal bovine serum (FBS), penicillin–streptomycin, and trypsin–EDTA, were purchased from Gibco (Waltham, MA, USA). Liquid chromatography-grade acetonitrile was obtained from Merck (Darmstadt, Germany), while formic acid was purchased from Sigma-Aldrich (Burlington, MA, USA). The reference compounds 3,4-dihydroxybenzoic acid and luteolin were obtained from ChemFaces (Wuhan, China). Unless otherwise specified, all chemicals and reagents were of analytical grade or of a grade appropriate for their intended analyses.

### 5.2. Plant Materials

Stems, leaves, and flowers of *P. sirindhorniae* were collected in Bueng Kan Province, Thailand (18°07′41.1″ N, 103°52′35.1″ E). The plant material was taxonomically authenticated by Associate Professor Sirivan Athikomkulchai, Faculty of Pharmacy, Srinakharinwirot University, Nakhon Nayok, Thailand. A voucher specimen (SIRA009) was deposited at the Faculty of Pharmacy, Srinakharinwirot University.

### 5.3. Methods

#### 5.3.1. Plant Extraction

Dried plant materials were rinsed with distilled water and air-dried to remove surface impurities. The plant materials were then cut and ground into powders. Each sample (4 g) was macerated in 160 mL of 95% (*v*/*v*) ethanol and stirred on a magnetic stirrer at room temperature for 90 min. After the macerate was separated, the extraction was repeated by soaking the marc with 95% ethanol (100 mL) for 90 min to ensure complete extraction of soluble constituents. The combined filtrates were transferred to a round-bottom flask and concentrated under reduced pressure using a rotary evaporator (water-bath temperature 37.0 ± 0.1 °C) until the ethanol was completely removed. The remaining aqueous fractions of the stems, leaves, and flowers were further evaporated to dryness using an evaporator dish to obtain the crude extracts, designated as BSSE, BSLE, and BSFE, respectively.

#### 5.3.2. Determination of Total Phenolic Content

The total phenolic content (TPC) of BSFE, BSSE, and BSLE was determined using the Folin–Ciocalteu assay, with gallic acid (3.9–125 µg/mL) as the reference standard. Each extract or gallic acid solution (50 µL) was transferred to a 96-well plate and mixed with 100 µL of 10% (*v*/*v*) Folin–Ciocalteu reagent. After 4 min, 50 µL of 10% (*w*/*v*) sodium carbonate solution was added. The reaction mixtures were maintained in the dark for 60 min [31]. after which the absorbance was measured at 765 nm. TPC was quantified from the gallic acid calibration curve and reported as gallic acid equivalents (GAE).

#### 5.3.3. Determination of Total Flavonoid Content

The total flavonoid content (TFC) of BSFE, BSSE, and BSLE was determined using an aluminum chloride colorimetric assay, with EGCG (7.8–500 µg/mL) as the reference standard. Each extract or EGCG solution (50 µL) was transferred to a 96-well plate and mixed with 30 µL of 5% (*w*/*v*) sodium nitrate solution. Following incubation for 5 min, 50 µL of 2% (*w*/*v*) aluminum chloride solution was added, and the reaction was allowed to proceed for an additional 6 min. Subsequently, 50 µL of 1 N sodium hydroxide was added, and the mixture was incubated for 10 min. Absorbance was measured at 510 nm [31]. TFC was calculated from the EGCG calibration curve and expressed as EGCG equivalents (EGCGE).

#### 5.3.4. DPPH Radical Scavenging Assay

The free radical scavenging capacity of BSFE, BSSE, and BSLE was assessed using the DPPH assay with minor modifications to a previously reported method. Aliquots (100 µL) of each extract at concentrations ranging from 3.9 to 2000 µg/mL were mixed with an equal volume of 0.1 mM DPPH solution in a 96-well microplate. The reaction mixtures were kept at room temperature in the dark for 30 min, after which absorbance was recorded at 517 nm using a microplate reader. Gallic acid served as the positive control. The percentage of DPPH radical scavenging activity was calculated as follows:

$$\mathrm{DPPH}\;\mathrm{Scavenging}\;(\%)=(1-\frac{A_{\mathrm{sample}}}{A_{\mathrm{control}}})\times 100$$

where $A_{\mathrm{sample}}$ is the absorbance of the reaction mixture containing the extract, and $A_{\mathrm{control}}$ is the absorbance of the DPPH solution without the extract. The concentration required to inhibit 50% of DPPH radicals (IC_50_) was calculated from the concentration-response curve using GraphPad Prism version 8.0.

#### 5.3.5. ABTS Radical Scavenging Assay

The ABTS radical cation scavenging activity of BSFE, BSSE, and BSLE was determined using a previously reported method with minor modifications. Aliquots (20 µL) of each extract at concentrations ranging from 3.9 to 2000 µg/mL were added to a 96-well microplate and mixed with 180 µL of freshly prepared 0.1 mM ABTS^•+^ solution. The reaction mixtures were incubated at room temperature in the dark for 15 min, after which absorbance was recorded at 734 nm using a microplate reader. Gallic acid served as the positive control. The percentage of ABTS radical scavenging activity was calculated as follows:

$$\mathrm{ABTS}\;\mathrm{Scavenging}\;(\%)=(1-\frac{A_{\mathrm{sample}}}{A_{\mathrm{control}}})\times 100$$

where $A_{\mathrm{sample}}$ is the absorbance of the reaction mixture containing the extract and $A_{\mathrm{control}}$ is the absorbance of the ABTS^•+^ solution without the extract. The IC_50_ value was calculated from the concentration–response curve using GraphPad Prism version 8.0.

#### 5.3.6. Ferric Reducing Antioxidant Power (FRAP) Assay

The ferric reducing capacity of BSFE, BSSE, and BSLE was assessed using the FRAP assay with minor modifications to a previously reported method. The FRAP working reagent was freshly prepared by combining 300 mM acetate buffer (pH 3.6), 10 mM TPTZ solution, and 20 mM ferric chloride solution at a ratio of 10:1:1 (*v*/*v*/*v*). Each extract (20 µL) was mixed with 180 µL of the FRAP reagent in a 96-well microplate and incubated at 37 °C for 30 min. Absorbance was subsequently recorded at 595 nm using a SpectraMax M3 microplate reader (Molecular Devices, San Jose, CA, USA). Gallic acid was included as a reference antioxidant. Ferric reducing capacity was quantified using a ferrous sulfate calibration curve (9.8–5000 µM) and expressed as Fe^2+^ equivalents.

#### 5.3.7. Cell Culture

HaCaT human keratinocytes and RAW264.7 murine macrophages were maintained in high-glucose DMEM supplemented with 10% (*v*/*v*) FBS and 1% (*v*/*v*) penicillin–streptomycin. Cells were cultured at 37 °C in a humidified atmosphere containing 5% CO_2_. HaCaT cells were subcultured as required using 0.25% trypsin–EDTA.

#### 5.3.8. Cytotoxicity Evaluation

The cytotoxic effects of BSFE, BSSE, and BSLE were assessed using the MTT assay. HaCaT keratinocytes and RAW264.7 macrophages were seeded separately into 96-well plates at a density of 8 × 10^3^ cells/well and incubated for 24 h. The cells were subsequently exposed to BSFE, BSSE, or BSLE at concentrations ranging from 1.95 to 1000 µg/mL for an additional 24 h. Cells maintained in complete culture medium without extract treatment were used as the untreated control.

Following treatment, the medium was removed and MTT solution (0.5 mg/mL) was added. After incubation at 37 °C for 2 h, the resulting formazan crystals were solubilized with DMSO (100 µL/well). Absorbance was measured at 550 nm using a SpectraMax M3 microplate reader (Molecular Devices, San Jose, CA, USA). Cell viability was calculated according to the following equation:

$$\mathrm{Cell}\;\mathrm{Viability}\;(\%)=\frac{A_{550\;\mathrm{sample}}}{A_{550\;\mathrm{control}}}\times 100$$

where $A_{\mathrm{sample}}$ is the absorbance of treated cells and $A_{\mathrm{control}}$ is the absorbance of untreated control cells.

The half-maximal inhibitory concentration (IC_50_) values were determined by nonlinear regression analysis using GraphPad Prism version 8.0 (GraphPad Software, San Diego, CA, USA).

#### 5.3.9. Nitric Oxide (NO) Production Assay

The effects of BSFE, BSSE, and BSLE on NO production were investigated in LPS-stimulated RAW264.7 macrophages using the Griess assay. Cells were seeded into 96-well plates at a density of 2 × 10^3^ cells/well and cultured for 24 h. The cells were then pretreated with 75 µL of each extract at non-cytotoxic concentrations in serum-free DMEM for 3 h. L-NAME (200 µM), a nitric oxide synthase inhibitor, was included as the positive control.

Following pretreatment, 75 µL of LPS solution (10 µg/mL) was added to obtain a final LPS concentration of 5 µg/mL, and the cells were incubated for a further 24 h. Culture supernatant (100 µL) was subsequently transferred to a new 96-well plate for nitrite determination. The Griess reagents were freshly prepared as follows: sulfanilamide (250 mg) was dissolved in 25 mL of 5% phosphoric acid, and N-(1-naphthyl)ethylenediamine dihydrochloride (NED; 25 mg) was dissolved separately in 25 mL of deionized water. The supernatant was sequentially mixed with 50 µL of the sulfanilamide solution and 50 µL of the NED solution, then incubated at room temperature in the dark for 10 min. Absorbance was recorded at 540 nm using a SpectraMax M3 microplate reader (Molecular Devices, San Jose, CA, USA). Nitrite concentrations were determined from a sodium nitrite calibration curve (0.59–300 µM), and the results were expressed relative to those of the LPS-stimulated control.

#### 5.3.10. Quantification of PGE_2_, TNF-α, IL-1β, and IL-6 by ELISA

The effects of the extracts on inflammatory mediator production were evaluated in LPS-stimulated RAW264.7 macrophages. Cells were seeded into 96-well plates at a density of 2 × 10^3^ cells/well and cultured for 24 h. After removal of the culture medium, cells were pretreated with 100 µL of serum-free DMEM containing the test extracts at non-cytotoxic concentrations for 3 h at 37 °C under 5% CO_2_. Cells receiving serum-free DMEM without test extract served as controls, while BAY 11-7082 (10 µM), an NF-κB inhibitor, was used as the positive control. The inflammatory response was subsequently induced with LPS at a final concentration of 5 µg/mL, followed by incubation for 24 h. Culture supernatants were collected, and the concentrations of PGE_2_, TNF-α, IL-1β, and IL-6 were determined using the respective commercial ELISA kits in accordance with the manufacturers’ protocols.

#### 5.3.11. Antimicrobial Assay

The antimicrobial activities of BSSE, BSFE, and BSLE were evaluated using the broth microdilution method [32]. Serial dilutions of each extract were prepared over the concentration range of 32–1024 µg/mL and tested against *Escherichia coli* ATCC 25922, *Pseudomonas aeruginosa* ATCC 27853, *Staphylococcus aureus* ATCC 25923, and *Staphylococcus epidermidis* ATCC 35984. Gentamicin was used as the positive control for the Gram-negative bacteria, whereas vancomycin was used for the Gram-positive bacteria. The minimum inhibitory concentration (MIC) was defined as the lowest concentration that completely inhibited visible bacterial growth. The minimum bactericidal concentration (MBC) was subsequently determined using the drop-plate method and was defined as the lowest concentration at which no bacterial colonies were observed on tryptic soy agar (TSA) plates.

#### 5.3.12. HPLC Analysis of 3,4-Dihydroxybenzoic Acid and Luteolin in BSLE

##### Chromatographic Conditions

HPLC analysis was performed using an Agilent 1260 Infinity II system (Agilent Technologies, Santa Clara, CA, USA) equipped with a quaternary pump, autosampler, multi-column thermostat, and photodiode array (PDA) detector. Chromatographic separation was achieved on an ACE 5 C18-AR column (4.6 × 250 mm, 5 µm) fitted with a Phenomenex C_18_ guard column (4 × 3 mm, 5 µm). The column temperature was maintained at 25 °C. The mobile phase consisted of 0.1% (*v*/*v*) formic acid in ultrapure water (A) and 0.1% (*v*/*v*) formic acid in acetonitrile (B). Gradient elution was performed as follows: 0–10 min, 2% B; 10–30 min, 2–25% B; 30–55 min, 25–90% B; 55–60 min, 90% B; 60–61 min, 90–2% B; and 61–71 min, 2% B for column re-equilibration. The flow rate was set at 1.0 mL/min, and the injection volume was 10 µL. Chromatograms were monitored at 295 nm.

##### Standard Solution Preparation

Individual stock solutions of 3,4-dihydroxybenzoic acid and luteolin were prepared in ethanol at a concentration of 200 µg/mL. Working standard solutions were subsequently prepared by appropriate dilution of the respective stock solutions to obtain concentration ranges of 18–90 µg/mL for 3,4-dihydroxybenzoic acid and 2–10 µg/mL for luteolin. Calibration curves were constructed by plotting chromatographic peak area against the corresponding standard concentration.

##### Sample Solution Preparation

BSLE (50 mg) was accurately weighed and dissolved in 10 mL of ethanol to obtain a final concentration of 5 mg/mL. Prior to HPLC analysis, the sample and standard solutions were passed through 0.45 µm nylon membrane filters.

#### 5.3.13. Preparation of BSLE-Loaded Hydrogel Sheets

Two BSLE-loaded hydrogel sheet formulations (BSLE-F1 and BSLE-F2) were developed using hydroxypropyl methylcellulose (HPMC) as the primary matrix former, together with polyvinyl alcohol (PVA), glycerol, PEG 600, and ethanol as auxiliary excipients. First, HPMC (8 g) was gradually dispersed in 50 mL of hot distilled water (70–80 °C) under continuous stirring until a clear, viscous solution was obtained. In parallel, a PVA solution was prepared in hot water and combined with glycerol (3.44 mL) to enhance flexibility. This PVA–glycerol mixture (46.56 mL) was subsequently incorporated into the HPMC dispersion, producing a homogeneous hydrogel base.

To incorporate the extract, BSLE extract (0.5 g) and PEG 600 were dissolved in 14.6 mL of 95% ethanol with gentle heating until fully solubilized. The PEG 600 content varied between formulations: BSLE-F1 contained 2 g PEG, and BSLE-F2 contained 6 g PEG. The warm ethanolic extract mixture was slowly added to the hydrogel base under continuous mixing to ensure uniform distribution, yielding a smooth gelling solution. The final solution was poured into glass Petri dishes lined with baking paper while still warm, then spread to achieve an even thickness. The dishes were subsequently cooled in a cold-water bath until the sheet became clear. The hydrogel sheets were dried in a hot-air oven at 45 °C for 1 h to facilitate solvent evaporation, then conditioned at room temperature for 30 min. Fully dried sheets were carefully removed from the backing.

#### 5.3.14. Characterization of BSLE-Loaded Hydrogel Sheets

Two BSLE-loaded hydrogel sheet formulations (BSLE-F1 and BSLE-F2) were characterized for appearance, pH, water-loss index, thickness, washability, peelability, swelling behavior, and mechanical properties [33]. All measurements were performed in triplicate, and results were expressed as mean ± standard deviation.

##### Visual Appearance

The dried hydrogel sheets were visually inspected immediately after preparation (Day 0). Each hydrogel sheet was examined under ambient laboratory light for color, uniformity, transparency/opacity, surface smoothness, flexibility, and tackiness. The presence of visible air bubbles, cracks, or surface defects was also recorded. The macroscopic appearance of BSLE-F1 and BSLE-F2 was compared descriptively.

##### pH Measurement

The pH of the hydrogel sheets was determined on Day 0 (before and after oven-drying) and after 7 days of storage at 4, 30, and 45 °C. Briefly, each sheet was cut into small pieces and dispersed in distilled water to obtain a 10% *w*/*v* dispersion. The mixture was allowed to equilibrate for 1 h with gentle stirring at room temperature. The pH was then measured using a calibrated digital pH meter at 25 ± 2 °C. The pH values of both formulations stored at different temperatures were recorded and compared.

##### Water Loss Index

The water-loss behavior of the hydrogel sheets was evaluated by monitoring changes in sheet weight during storage. The sheets were weighed after oven-drying (Day 0) to obtain the initial weight (*W*_0_). The sheets were then stored at 4, 30, or 45 °C in closed containers for 7 days. At Day 7, the sheets were reweighed to obtain the final weight (*W*_7_). The percentage water loss (%WL) was calculated using the equation:

$$\%\mathrm{Water}\;\mathrm{loss}=\frac{W_{0}-W_{7}}{W_{0}}\times 100$$

where *W*_0_ is the initial sheet weight, and *W*_7_ is the sheet weight after storage. The water-loss indices of BSLE-F1 and BSLE-F2 were compared under each storage condition.

##### Hydrogel Sheet Thickness

BSLE-loaded hydrogel sheet thickness was measured on Day 0 and Day 7 using a caliper vernier. For each formulation, three sheets were randomly selected, and the thickness was measured at three points (the center and two peripheral points) on each sheet. The mean thickness for each sheet and the overall mean ± SD for each formulation were calculated. The change in thickness (Δ thickness) after storage was calculated as the difference between the Day 7 and Day 0 thicknesses at each measurement point.

##### Washability

Washability was evaluated by determining the time required to completely remove residual hydrogel from the application surface with water. A known amount of each BSLE-loaded hydrogel formulation was applied to the test surface and left in place for 1 h. The hydrogel sheet was then peeled off, and the remaining residue was washed with water while the washing time was recorded using a stopwatch. Measurements were performed immediately after preparation (Day 0) and after 7 days of storage at 4, 30, and 45 °C. The time required for complete removal of visible residual hydrogel was recorded in seconds. All measurements were performed in triplicate, and the results were expressed as mean ± standard deviation.

##### Peelability and Residual Gel on Substrate

Peelability was assessed indirectly by measuring the amount of gel remaining on a plastic substrate after removal. A known amount of hydrogel was cast or pressed onto pre-weighed plastic plates and allowed to stand for 1 h to mimic application on the skin. The hydrogel sheet was then manually peeled off. The plates were reweighed to obtain the final weight (*W*_plate,after_). The percentage of residual gel remaining on the substrate (%Gel residue) was calculated relative to the weight of the empty plate (*W*_plate,blank_) and the initial applied gel, as follows:

$$\%\mathrm{Gel}\;\mathrm{residue}=\frac{W_{\mathrm{plate},\mathrm{after}}-W_{\mathrm{plate},\mathrm{blank}}}{W_{\mathrm{initial}\;\mathrm{gel}}}\times 100$$

Lower residue percentages were interpreted as better peelability. BSLE-F1 and BSLE-F2 were compared based on their mean gel-residue percentages.

##### Swelling Index

Swelling behavior was evaluated by immersing the dried hydrogel sheets in distilled water. BSLE-loaded hydrogel sheets were cut into uniform pieces, dried to constant weight, and weighed to obtain the initial dry weight (*W*_dry_). Each sheet was then placed in a beaker containing an excess of distilled water at room temperature. At predetermined time intervals (5, 15, and 30 min), the sheets were removed, gently blotted with filter paper to remove surface water, and immediately weighed to obtain the swollen weight (*W*_*t*_). The swelling index (SI) was calculated using:

$$\mathrm{Swelling}\;\mathrm{index}\;(\%)=\frac{W_{t}-W_{\mathrm{dry}}}{W_{\mathrm{dry}}}\times 100$$

The swelling profiles of BSLE-F1 and BSLE-F2 were compared over time.

##### Mechanical Properties

The tensile strength of the hydrogel sheets was assessed using a texture analyzer equipped with a tensile grip set. Hydrogel sheets of each formulation were cut into strips (2 × 5 cm^2^). The initial width and thickness of each strip were measured using a digital micrometer. The sheet strips were mounted between the upper and lower clamps to ensure a uniform gauge length. The sheets were stretched at a constant crosshead speed until break, and the maximum force at break (F, N) was recorded. Tensile strength (TS) was calculated using:

$$\mathrm{Tensile}\;\mathrm{strength}\;({N/\mathrm{mm}}^{2})=\frac{F}{\mathrm{Width}\;(\mathrm{mm})\times \mathrm{Thickness}\;(\mathrm{mm})}$$

The mean tensile strength values for each formulation were used to compare the mechanical robustness of BSLE-F1 and BSLE-F2.

##### Statistical Analysis

All measurements were conducted in three independent replicates, and the data are presented as mean ± standard deviation (SD). Statistical evaluation was performed using GraphPad Prism version 8.0 (GraphPad Software, San Diego, CA, USA). Differences among three or more groups were assessed by one-way analysis of variance (ANOVA) followed by Tukey’s multiple-comparisons test, whereas comparisons between two groups were performed using an unpaired *t*-test. Statistical significance was defined as *p* < 0.05.

## Data Availability

The data presented in this study are available within the article.

## References

[1] Gonfa Y.H., Tessema F.B., Bachheti A., Rai N., Tadesse M.G., Nasser Singab A., Chaubey K.K., Bachheti R.K. (2023). Anti-inflammatory activity of phytochemicals from medicinal plants and their nanoparticles: A review. Curr. Res. Biotechnol..

[2] Mackinder B., Clark R. (2014). A synopsis of the Asian and Australasian genus *Phanera* Lour. (Cercideae: Caesalpinioideae: Leguminosae) including 19 new combinations. Phytotaxa.

[3] Jirukkakul N., Poolklang A. (2024). Utilization of *Bauhinia sirindhorniae* leaves extract to improve the antioxidant property of gelatin/sarcoplasmic protein film. Food Appl. Biosci. J..

[4] Athikomkulchai S. (2004). Chemical Constituents and Biological Activities of *Bauhinia sirindhorniae* and Croton Hutchinsonianus. Ph.D. Thesis.

[5] Athikomkulchai S., Sriubolmas N., Ruangrungsi N. (2019). Antibacterial Activity of Flavonoids from *Bauhinia sirindhorniae*. J. Health Res..

[6] Nithikulworawong N. (2012). Antibacterial Activity of *Bauhinia sirindhorniae* Extract Against *Aeromonas hydrophila* Isolated from Hybrid catfish. Walailak J. Sci. Technol..

[7] Fernandes A., Rodrigues P.M., Pintado M., Tavaria F.K. (2023). A systematic review of natural products for skin applications: Targeting inflammation, wound healing, and photo-aging. Phytomedicine.

[8] Farhan M. (2024). The Promising Role of Polyphenols in Skin Disorders. Molecules.

[9] Musuc A.M., Mititelu M., Chelu M. (2024). Hydrogel for Sustained Delivery of Therapeutic Agents. Gels.

[10] Segneanu A.-E., Bejenaru L.E., Bejenaru C., Blendea A., Mogoşanu G.D., Biţă A., Boia E.R. (2025). Advancements in Hydrogels: A Comprehensive Review of Natural and Synthetic Innovations for Biomedical Applications. Polymers.

[11] Liu B., Chen K. (2024). Advances in Hydrogel-Based Drug Delivery Systems. Gels.

[12] Sharma B., Rani J., Vc V., Sable P., Kumar S., Marndi S., Das K. (2025). Bioactive Compounds of Medicinal Plants: Structure, Function, and Bioactivity. J. Biodivers. Conserv..

[13] Leopoldini M., Marino T., Russo N., Toscano M. (2004). Antioxidant Properties of Phenolic Compounds: H-Atom versus Electron Transfer Mechanism. J. Phys. Chem. A.

[14] Song F.L., Gan R.Y., Zhang Y., Xiao Q., Kuang L., Li H.B. (2010). Total phenolic contents and antioxidant capacities of selected chinese medicinal plants. Int. J. Mol. Sci..

[15] Spiridon I., Bodirlau R., Teaca C.-A. (2011). Total phenolic content and antioxidant activity of plants used in traditional Romanian herbal medicine. Cent. Eur. J. Biol..

[16] Khiya Z., Oualcadi Y., Gamar A., Berrekhis F., Zair T., Hilali F.E.L. (2021). Correlation of Total Polyphenolic Content with Antioxidant Activity of Hydromethanolic Extract and Their Fractions of the *Salvia officinalis* Leaves from Different Regions of Morocco. J. Chem..

[17] El-Saadony M.T.T., Saad A.M.M., Mohammed D.M., Alkafaas S.S.S., Abd El-Mageed T.A.A., Fahmy M.A., Ezzat Ahmed A., Algopishi U.B., Abu-Elsaoud A.M., Mosa W.F.A. (2025). Plant bioactive compounds: Extraction, biological activities, immunological, nutritional aspects, food application, and human health benefits—A comprehensive review. Front. Nutr..

[18] Merecz-Sadowska A., Sitarek P., Śliwiński T., Zajdel R. (2020). Anti-Inflammatory Activity of Extracts and Pure Compounds Derived from Plants via Modulation of Signaling Pathways, Especially PI3K/AKT in Macrophages. Int. J. Mol. Sci..

[19] Semaming Y., Pannengpetch P., Chattipakorn S.C., Chattipakorn N. (2015). Pharmacological properties of protocatechuic Acid and its potential roles as complementary medicine. Evid. Based Complement. Altern. Med..

[20] Aziz N., Kim M.Y., Cho J.Y. (2018). Anti-inflammatory effects of luteolin: A review of in vitro, in vivo, and in silico studies. J. Ethnopharmacol..

[21] De Rossi L., Rocchetti G., Lucini L., Rebecchi A. (2025). Antimicrobial Potential of Polyphenols: Mechanisms of Action and Microbial Responses—A Narrative Review. Antioxidants.

[22] Sutradhar S.C., Shin H., Kim W., Jang H. (2025). Hydrogel Films in Biomedical Applications: Fabrication, Properties and Therapeutic Potential. Gels.

[23] Frederiksen K., Guy R.H., Petersson K. (2015). Formulation considerations in the design of topical, polymeric film-forming systems for sustained drug delivery to the skin. Eur. J. Pharm. Biopharm..

[24] Owusu-Ware S.K., Boateng J.S., Chowdhry B.Z., Antonijevic M.D. (2019). Glassy state molecular mobility and its relationship to the physico-mechanical properties of plasticized hydroxypropyl methylcellulose (HPMC) films. Int. J. Pharm. X.

[25] Christoforou I., Kalatzis A., Siamidi A., Vlachou M., Pispas S., Pippa N. (2025). The Ubiquitous Use of Polyethylene Glycol in Pharmaceutical Design and Development: Technological Aspects and Future Perspectives. Nanomaterials.

[26] Saringat H., Alfadol K., Khan G.M. (2005). The influence of different plasticizers on some physical and mechanical properties of hydroxypropyl methylcellulose free films. Pak. J. Pharm. Sci..

[27] Rabek C., Stelle R., Dziubla T., Puleo D. (2014). The Effect of Plasticizers on the Erosion and Mechanical Properties of Polymeric Films. J. Biomater. Appl..

[28] Gounden V., Singh M. (2024). Hydrogels and Wound Healing: Current and Future Prospects. Gels.

[29] Joshi S.C. (2011). Sol-Gel Behavior of Hydroxypropyl Methylcellulose (HPMC) in Ionic Media Including Drug Release. Materials.

[30] Singer C.A., Abdul-Karim H., Printon K., Poluri N., Teng T., Akbari M., Modanloo B., Mogas-Soldevila L., Akbarzadeh M., Hu X. (2025). Plasticized agarose films: A physicochemical, mechanical and thermal study. Int. J. Biol. Macromol..

[31] Chittasupho C., Chaobankrang K., Sarawungkad A., Samee W., Singh S., Hemsuwimon K., Okonogi S., Kheawfu K., Kiattisin K., Chaiyana W. (2023). Antioxidant, Anti-Inflammatory and Attenuating Intracellular Reactive Oxygen Species Activities of *Nicotiana tabacum* var. Virginia Leaf Extract Phytosomes and Shape Memory Gel Formulation. Gels.

[32] Phongpradist R., Chittasupho C., Singh S., Ontong J.C., Tadtong S., Akachaipaibul P., Punvittayagul C., Thongkorn K., Dejkriengkraikul P., Jiaranaikulwanitch J. (2025). Investigation of a Thermoresponsive In Situ Hydrogel Loaded with Nanotriphala: Implications for Antioxidant, Anti-Inflammatory, and Antimicrobial Therapy in Nasal Disorders. Gels.

[33] Mutar M.T., Mahdee A.F. (2025). Characterization of a Hydrogel Composite Containing Bioactive *Moringa* as a Novel Pulp-Capping Material. Polymers.

